# A Novel Trehalose Synthase for the Production of Trehalose and Trehalulose

**DOI:** 10.1128/Spectrum.01333-21

**Published:** 2021-11-24

**Authors:** Neera Agarwal, Sudhir P. Singh

**Affiliations:** a Center of Innovative and Applied Bioprocessing, Mohali, India; b Department of Biotechnology, Panjab University, Chandigarh, India; University of Minnesota

**Keywords:** trehalose, trehalulose, trehalose synthase, metagenome, low-cost sucrose feedstocks, metagenome

## Abstract

A novel putative trehalose synthase gene (*treM*) was identified from an extreme temperature thermal spring. The gene was expressed in Escherichia coli followed by purification of the protein (TreM). TreM exhibited the pH optima of 7.0 for trehalose and trehalulose production, although it was functional and stable in the pH range of 5.0 to 8.0. Temperature activity profiling revealed that TreM can catalyze trehalose biosynthesis in a wide range of temperatures, from 5°C to 80°C. The optimum activity for trehalose and trehalulose biosynthesis was observed at 45°C and 50°C, respectively. A catalytic reaction performed at the low temperature of 5°C yielded trehalose with significantly reduced by-product (glucose) production in the reaction. TreM displayed remarkable thermal stability at optimum temperatures, with only about 20% loss in the activity after heat (50°C) exposure for 24 h. The maximum bioconversion yield of 74% trehalose (at 5°C) and 90% trehalulose (at 50°C) was obtained from 100 mM maltose and 70 mM sucrose, respectively. TreM was demonstrated to catalyze trehalulose biosynthesis utilizing the low-cost feedstock jaggery, cane molasses, muscovado, and table sugar.

**IMPORTANCE** Trehalose is a rare sugar of high importance in biological research, with its property to stabilize cell membrane and proteins and protect the organism from drought. It is instrumental in the cryopreservation of human cells, e.g., sperm and blood stem cells. It is also very useful in the food industry, especially in the preparation of frozen food products. Trehalose synthase is a glycosyl hydrolase 13 (GH13) family enzyme that has been reported from about 22 bacterial species so far. Of these enzymes, to date, only two have been demonstrated to catalyze the biosynthesis of both trehalose and trehalulose. We have investigated the metagenomic data of an extreme temperature thermal spring to discover a novel gene that encodes a trehalose synthase (TreM) with higher stability and dual transglycosylation activities of trehalose and trehalulose biosynthesis. This enzyme is capable of catalyzing the transformation of maltose to trehalose and sucrose to trehalulose in a wide pH and temperature range. The present investigation endorses the thermal aquatic habitat as a promising genetic resource for the biocatalysts with high potential in producing high-value rare sugars.

## INTRODUCTION

Trehalose is a nonreducing sugar, comprised of two glucose units linked via an α,α-(1) glycosidic bond (1). Edible mushrooms contain a high trehalose content; therefore, it is also known as mycose or mushroom sugar (2). It occurs in a wide range of organisms, e.g., mycobacteria, yeasts, insects, nematodes, and plants (2). Although trehalose has been detected in many vertebrates, it could not be traced in mammalian cells (1). Scientific studies have identified several biological functions of trehalose, e.g., protection of the organism against drought as a water replacement or vitrification agent; stabilizer of the cell membrane and proteins; antistress agent during hypothermia, hyperthermia, and osmotic stress; source of energy in insects that could be consumed during flight; and as a cell wall component in mycobacteria and corynebacteria (1).

Trehalose had been accorded the status of Generally Recognized as Safe (GRAS) by US Food Drug Administration in 2000 (FDA, GRN no. 000045) (3). It has 45% sweetness compared with that of sucrose (4) and is less cariogenic (5). However, its caloric value is similar to that of sucrose, i.e., 4 kcal/g (6). Trehalose is a highly stable sugar in a wide range of pHs that could be due to low energy glycosidic bonds compared with other disaccharides (1). With these properties, trehalose is used as a preferred additive for flavor enhancement in the food industry sector (3). It prevents starch aging (7) and is helpful in extending the shelf-life of a food product. It lowers the freezing point of the food products (3, 8). In Japan, it is extensively used in confectionery and in the preparation of frozen food products, including ice creams (3, 9). Trehalose was found a potent ingredient that could diminish the progression of insulin resistance (10). Trehalose had also been reported to maintain the polyphenolic content and antioxidant properties of food products (11). It has a high water retention capability, preventing water content loss in the products of food, pharmaceutical, and cosmetic industries (12). In the pharmaceutical industry, trehalose is used for the cryopreservation of sperm and blood stem cells, without affecting cell viability (13, 14). Furthermore, trehalose stabilizes the protein structures (15) and reduces peptide aggregation (16). It is a promising agent for the preservation of various tissues and organs for transplantation (17). In the cosmetic industry, trehalose is used as a storage stability enhancer, moisture-retaining agent, and suppressor of foul odor in creams and lotions (3). It also helps in reducing the human body odor by degrading unsaturated fatty acids (4).

Trehalose can be synthesized chemically via the ethylene oxide addition reaction between 2,3,4,5-tetra-*O*-acetyl-d-glucose and 3,4,6-tri-*O*-acetyl-1,2-anhydro-d-glucose (18). However, the chemical synthesis of trehalose is very costly (1). The biological route of trehalose production is possible by employing microbial enzyme systems. The enzymatic process of trehalose synthesis is preferred due to its low-cost and more straightforward method. Primarily, trehalose biosynthesis is performed via three biocatalytic pathways, comprising different enzymes, as follows: (i) trehalose-6-phosphate synthase and trehalose-6-phosphate phosphatase, (ii) maltooligosyl trehalose synthase and maltooligosyl trehalose trehalohydrolase, and (iii) trehalose synthase (19). Trehalose synthase executes the reversible conversion reaction of maltose to trehalose. The trehalose synthase-based process is relatively simple and economical (20). This enzyme may evince both transglucosidic as well as hydrolytic activities. Besides trehalose biosynthesis, it can catalyze the conversion of sucrose into trehalulose. Trehalulose [α-d-glucopyranosyl-(l→l)-d-fructose] is a ketose analogue of trehalose, comprising glucose and fructose residues linked with an α-1,1 glycosidic bond. Trehalulose is a noncrystalline and noncariogenic sugar that occurs naturally in the honey of the stingless bee (21). The reduced glycemic index and antioxidant property suggest its possible use as a functional ingredient. It was also reported to be a potent antidiabetic and antioxidant agent (22). Being highly soluble in water, this sugar should be helpful in making jams and jellies.

Trehalose synthase is a glycosyl hydrolase 13 (GH13) family enzyme (23). For the first time, it was identified in *Pimelobacter* sp. R48 (24). Subsequently, it had been characterized from about 22 bacterial species (see Table S1 in the supplemental material). Out of these species, trehalose synthase from Thermomonospora curvata (23) and Thermus aquaticus (25) had been demonstrated to catalyze the biosynthesis of both trehalose and trehalulose. The present study reports the identification and characterization of a novel trehalose synthase (TreM) from the metagenome of an extreme temperature thermal spring for the biosynthesis of trehalose and trehalulose.

## RESULTS

### Gene sequence analysis and homology model.

**FIG 1:**
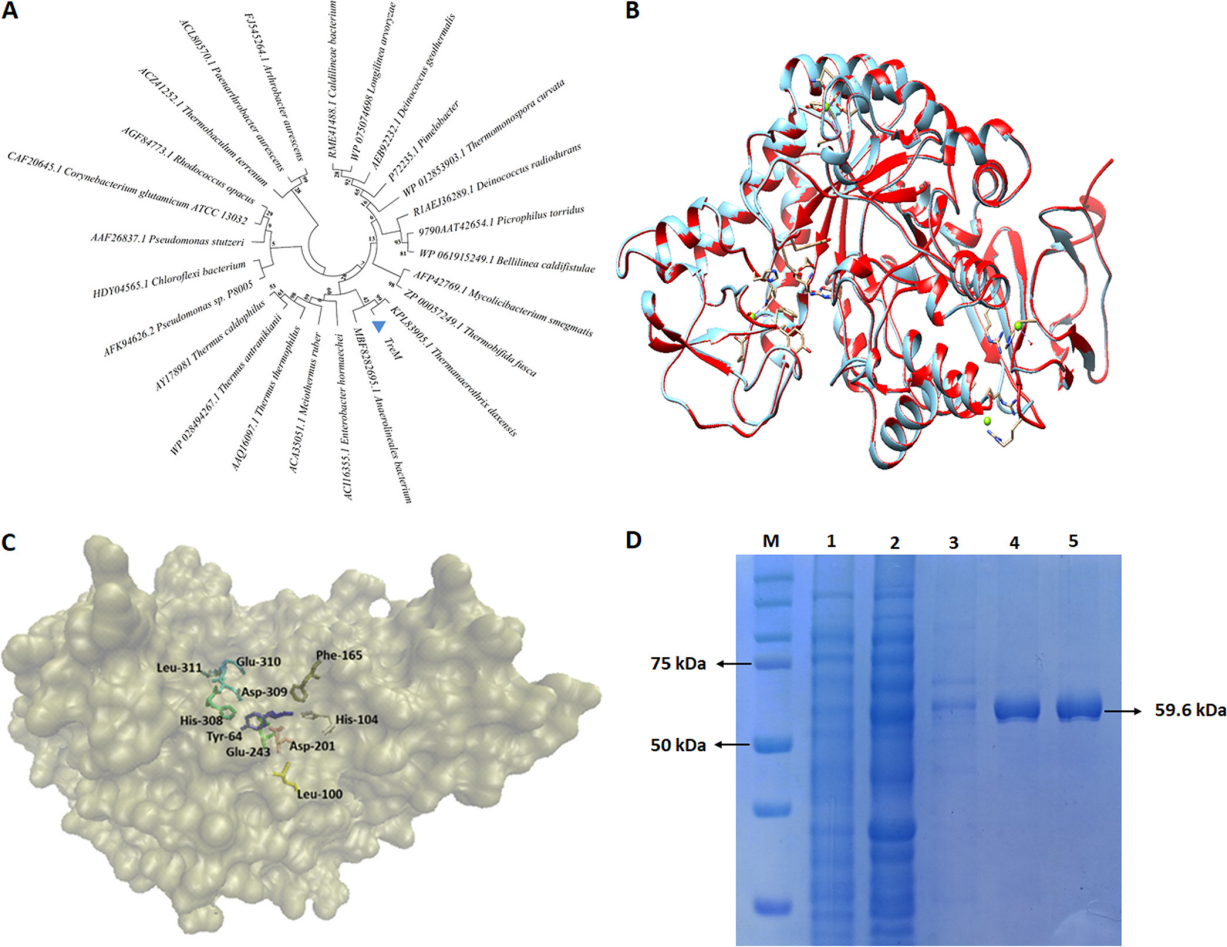
A dendrogram showing the phylogenetic relationship among the previously characterized trehalose synthases and TreM. The number mentioned at the points of branching indicate the bootstrap values (A). Superimposition of TreM (blue) over its template (red), i.e., trehalose synthase from *Thermobaculum terrenum*. The computed RMSD value was 0.075 Å (B). Homology model of TreM showing amino acid residues critical in substrate binding and catalytic action (C). SDS-PAGE showing protein bands (D); M, ladder; 1, cell-crude extract of vector-transformed E. coli; 2, cell-crude extract of TreM-expressed E. coli; 3, TreM eluted using 100 mM imidazole; 4 and 5, purified TreM eluted using 200 mM imidazole.

The putative trehalose synthase gene (*treM*) sequence, cloned from a thermal spring metagenome, was subjected to similarity analysis against the NCBI (nucleotide and protein) sequence databases. At the nucleotide level, *treM* was about 72.8% identical to a genomic sequence from Schlegelella thermodepolymerans, at 42% of query coverage. However, it exhibited about 88% identity at the protein level with an uncharacterized sequence from Thermanaerothrix daxensis in the NCBI protein database. TreM discerned a closer relatedness in the phylogenetic tree with the orthologous sequences from *Thermanaerothrix daxensis* and Anaerolineales bacterium (Fig. 1A). The maximum identity of about 58% was noted with a previously characterized trehalose synthase from Thermobaculum terrenum. A sequence comparison with trehalose synthases from Thermomonospora curvata and Thermus aquaticus, exhibiting dual catalytic function, divulged about 57% and 52% identity with TreM, respectively.

Mapping of TreM sequence to Conserved Domain Database (CDD) depicted an α-amylase domain, a typical feature of the glycosyl hydrolase 13 (GH13) family (Fig. S1A). Secondary structure analysis illustrated the presence of 31% α-helix and 18% β-sheets in TreM (Fig. S1B). A comparative sequence analysis with previously characterized trehalose synthases led to identifying three domains, namely, A, B, and C, in TreM. Domain A was predicted to adopt a triosephosphate isomerase (TIM) (β/α)_8_ barrel structure and an extended active site loop and, thus, forms the catalytic core of the enzyme. Domain B was typically an α-helix, with a calcium-binding site. Domain C was observed to have seven-stranded antiparallel β-sheets. Multiple sequence alignment among the previously characterized trehalose synthases revealed the conserved motifs in TreM, e.g., YYGDEIGMGDNI, LWLLP, DDGYDI, and DAVPYL (see Fig. S2 in the supplemental material).

A three-dimensional (3D) model of the TreM was built using the template sequences in Phyre2 software. The homology model was superimposed on the best hit template *Thermobaculum terrenum* trehalose synthase (PDB 5X7U), using Chimera 1.13, with a root mean square deviation (RMSD) value of 0.075 Å (Fig. 1B). The conserved residues critical for the catalytic activity were identified by sequence comparison and showed in the homology model of TreM. The five conserved residues, critical in constituting the catalytic pocket (His104, Asp201, Glu243, His308, and Asp309) (23), were identified in the TIM (β/α)_8_ barrel structure of TreM (Fig. 1C). The substrate-binding residues Asp309 and Leu311 and the residues Asp201 and Glu243 that are critical in catalytic function, as the nucleophile and acid/base catalyst, respectively, were determined (Fig. 1C). Additionally, L100 and E310 amino acid residues, demonstrated to be critical for substrate specificity and transglycosylation activity of the protein (23), were identified in TreM. The residues Tyr64 and Phe165 that are specific to binding with sucrose, the substrate for trehalulose synthesis, were also identified in TreM (26). The alignment of the protein sequences from the previously characterized trehalose synthases and TreM inscribed the conserveness of the aforementioned critical amino acid residues (Fig. S2). Superimposition of the homology modeled structures of the dual-function trehalose synthases from Thermomonospora curvata, Thermus aquaticus, and TreM let out the RMSD value of 0.969. The superimposed protein model showed the amino acid residues critical for substrate binding and catalytic function (see Fig. S6B in the supplemental material).

### Expression and purification of TreM.

TreM was expressed in a heterologous host, Escherichia coli. The enzyme was purified by performing Nickel-chelating nitrilotriacetic acid (Ni-NTA) affinity chromatography followed by dialysis to remove NaCl and imidazole contamination. SDS-PAGE analysis revealed a single band of protein of approximately 60 kDa, assessing the purity of about 95% (Fig. 1D).

### Catalytic reactivity of TreM with various substrates.

The reactivity of TreM was examined toward various substrates *viz.* maltose, sucrose, glucose, trehalose, maltotriose, trehalulose, palatinose, turanose, lactose, and lactulose. Thin-layer chromatography (TLC) analysis of the reaction products revealed the capability of TreM to catalyze the transformation of maltose and sucrose into trehalose and trehalulose, respectively (see Fig. S3 in the supplemental material). Furthermore, the bioconversion of maltose to trehalose was reversible. The reaction of TreM with glucose, maltotriose, lactose, and lactulose did not yield any product. Interestingly, TreM was specific to act with sucrose, as no product formation occurred with the sucrose isomers trehalulose, palatinose, and turanose (Fig. S3).

### Optimum pH and temperature.

**FIG 2:**
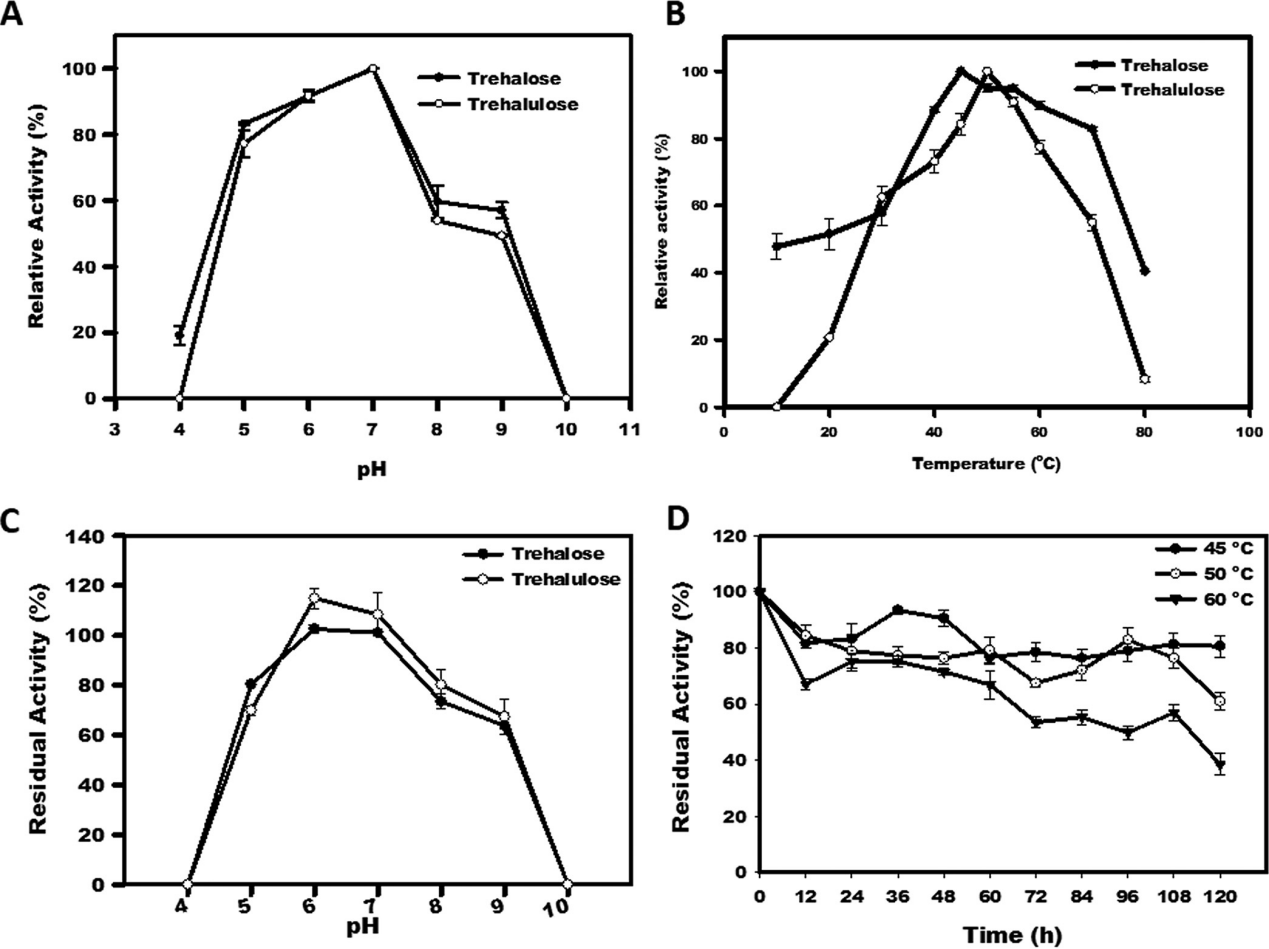
Effects of pH (A) and temperature (B) on the activity of TreM for the biosynthesis of trehalose and trehalulose. Residual activity of TreM exposed to 4.0 to 10.0 pH. The reaction performed using the enzyme fraction that was not pre-exposed to acidic or alkaline pH was considered control (C). Thermal stability of TreM at different temperatures (D).

The effect of pH on trehalose- and trehalulose-synthesizing activities of TreM was investigated in the acidic to alkaline pH (4.0 to 10.0), using maltose and sucrose as the substrate, respectively. The pH optima for the catalytic biosynthesis of trehalose or trehalulose was 7.0. In the case of trehalose synthesis, the relative activity of about 80% was recorded at the acidic pH of 5.0 and about 59% at a slightly alkaline pH (8.0) (Fig. 2A). However, the acidic pH of 4.0 was detrimental for both trehalose and trehalulose biosynthesis. Interestingly, the use of tris buffer (50 mM; pH 7.0) in the place of sodium phosphate buffer decreased the enzymatic activity of TreM by about 43%.

The relative activity (%) of TreM was recorded in the temperature range of 10°C to 80°C. The optimum temperature for trehalose synthesis was determined to be 45°C. However, TreM was found to be active at a low temperature of 10°C and a high temperature of 80°C and exhibited about 47% and 40% relative activity at these temperatures, respectively (Fig. 2B). For trehalulose biosynthesis, the favorable range of temperature was 40°C to 70°C, with optimal activity at 50°C (Fig. 2B).

### Thermal and pH stability of TreM.

After the heat exposure for 5 days, the enzyme retained about 80%, 60%, and 40% residual activities at 45°C, 50°C, and 60°C, respectively (Fig. 2D). The half-life of TreM was determined to be 96 h at 60°C. Thus, TreM displayed high thermal stability at optimum temperatures.

The aliquots of TreM were incubated in the buffers of different pHs for an hour. The enzyme displayed fairly good stability in the pH range of 5.0 to 8.0. The residual activity for trehalose and trehalulose synthesis was nil in the case of TreM exposed to pH 4.0 and 10.0 (Fig. 2C). The residual activity of TreM was significantly reduced in the buffer of pH 9.0, with the production of about 64% trehalose and 67% trehalulose (Fig. 2C).

### Effect of metal ions on TreM activity.

The presence of metal in the reaction, such as Ca^2+^, Fe^2+^, Na^+^, K^+^, Li^+^, Zn^2+^, Mn^2+^, and Mg^2+^, did not significantly affect the activity of TreM (Table 1). Furthermore, any notable change in the activity was not recorded in the presence of a chelating agent, EDTA, endorsing TreM as a metal-independent enzyme. However, Cu^2+^ was noticed to be detrimental, reducing the relative activity by about 30% and 60% for the catalytic synthesis of trehalose and trehalulose, respectively.

**TABLE 1:** Effect of metal ions and EDTA on the activity (relative activity [%] ± SD) of metagenomic trehalose synthase TreM

Treatment	Relative activity (%) of:	
	Trehalose	Trehalulose
Control	100 ± 0.04	100 ± 0.02
Co^2+^	84 ± 3.6	93 ± 8.5
Ca^2+^	92 ± 6.8	98 ± 5.8
Cu^2+^	72 ± 6.8	40 ± 4.7
Fe^2+^	90 ± 5.8	96 ± 0.4
Na^+^	94 ± 4.3	95 ± 0.3
K^+^	95 ± 3.2	93 ± 3.5
Li^+^	93 ± 1.7	92 ± 2.3
Zn^2+^	90 ± 3.9	86 ± 3.4
Mn^2+^	96 ± 4.7	121 ± 7.1
Mg^2+^	93 ± 4.7	104 ± 9.9
EDTA	93 ± 5.9	98 ± 1.4

### Effect of substrate and enzyme concentrations.

TreM catalysis for the biosynthesis of trehalose and trehalulose was examined in the presence of variable concentrations of substrates. A maltose-to-trehalose conversion of 60% to 70% was achieved in 1 to 3 h of the reaction performed with 100 to 700 mM substrate concentrations (see Fig. S4A in the supplemental material). The reaction conducted for a more extended period of time, i.e., 12 h or more, faced a relative decrease in the conversion yield of trehalose. In the case of trehalulose biosynthesis, the conversion yield (%) was about 87% in 12 h of the catalytic reaction with 70 mM sucrose. Interestingly, there was a sharp decrease in sucrose to trehalulose conversion (%), with the increasing concentration (100 mM to 1 M) of the substrate (Fig. S4B).

### Effect of glucose and fructose.

During maltose-to-trehalose conversion, glucose functions as an acceptor molecule in the transglycosylation reaction. The addition of glucose in the reaction medium negatively affected the TreM catalysis for the maltose-to-trehalose conversion (Fig. S4C). In contrast, the addition of fructose as an acceptor did not make any difference in the catalytic biosynthesis of trehalulose from sucrose (Fig. S4D).

### Catalytic production of trehalose at different temperatures.

**FIG 3:**
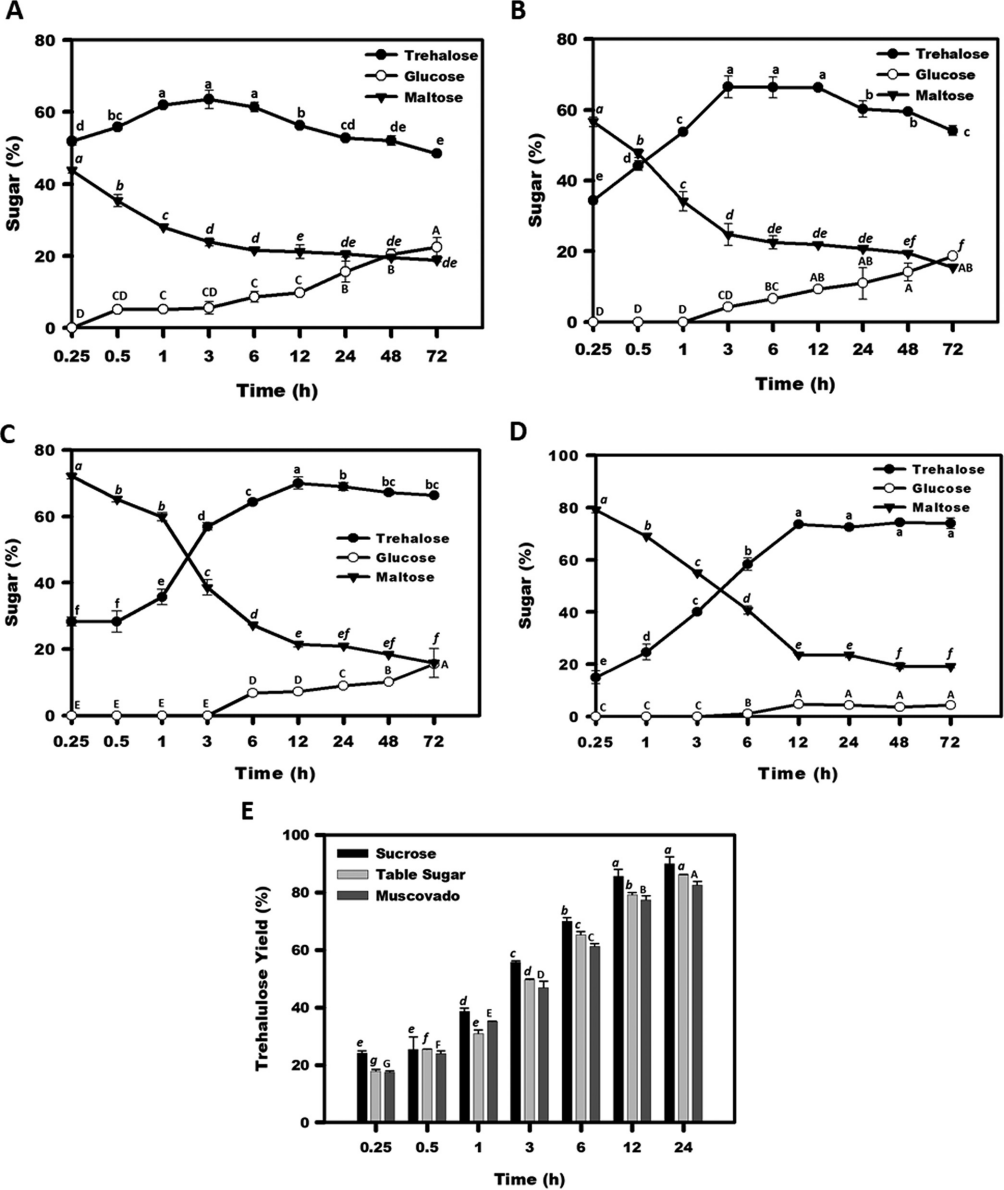
Maltose-to-trehalose conversion by the TreM catalytic reaction during different time points at 45°C (A), 30°C (B), 20°C (C), and 5°C (D). Production of trehalulose from sucrose, table sugar, and muscovado by the TreM catalytic reaction during different time points at 50°C (E). Mean values not sharing common alphabets in the assessment time points are statistically different at a *P* value of <0.05.

Catalytic bioconversion of maltose to trehalose was executed at different time points at the optimum temperature, i.e., 45°C. At this temperature, the rate of trehalose production was faster, achieving more than 52% bioconversion in 15 min (Fig. 3A). In a 3-h reaction, about 63% of bioconversion was achieved, which amounts to about 23 g · liter^−1^ trehalose yield, with generation of about 5% glucose as a by-product. The reactions for trehalose synthesis were also performed at lower temperatures, namely, 30°C (Fig. 3B), 20°C (Fig. 3C), and 5°C (Fig. 3D), to examine the trend of by-product formation in the cold reaction condition. At 30°C, the bioconversion yield of about 57% was attained in 1 h with nil by-product formation, while a similar trehalose yield could be achieved in a 3-h and 6-h reaction at 20°C and 5°C, respectively. However, low temperature (5°C) catalysis is preferable for trehalose biosynthesis due to a minimum production of by-product (27). In 12 h of a low-temperature catalysis reaction at 5°C, the conversion yield was about 74%, which amounts to the production of about 27 mg·mL^−1^ trehalose from 36.03 mg·mL^−1^ maltose monohydrate. Only 4% glucose (by-product) could be detected in 12 to 96 h of cold catalysis at 5°C.

### Trehalulose production.

The time course of the catalyzed reaction performed at optimum temperature (50°C) revealed a steady increase in sucrose to trehalulose bioconversion (%), until 24 h (Fig. 3E). The trehalulose yield of about 90% was achieved in 24 h of the enzymatic reaction, which amounts to the production of 21.5 g · liter^−1^ trehalulose. To further investigate the industrial use of TreM, low-cost sucrose feedstock *viz.* cane molasses, jaggery, muscovado, and table sugar were enzymatically treated. TLC analysis of the reaction products confirmed biosynthesis of trehalulose in TreM-treated feedstock (see Fig. S5 in the supplemental material). A trehalulose yield of 86% and 82% was achieved utilizing the feedstock table sugar and muscovado, respectively (Fig. 3E).

### Purification of reaction products.

**FIG 4:**
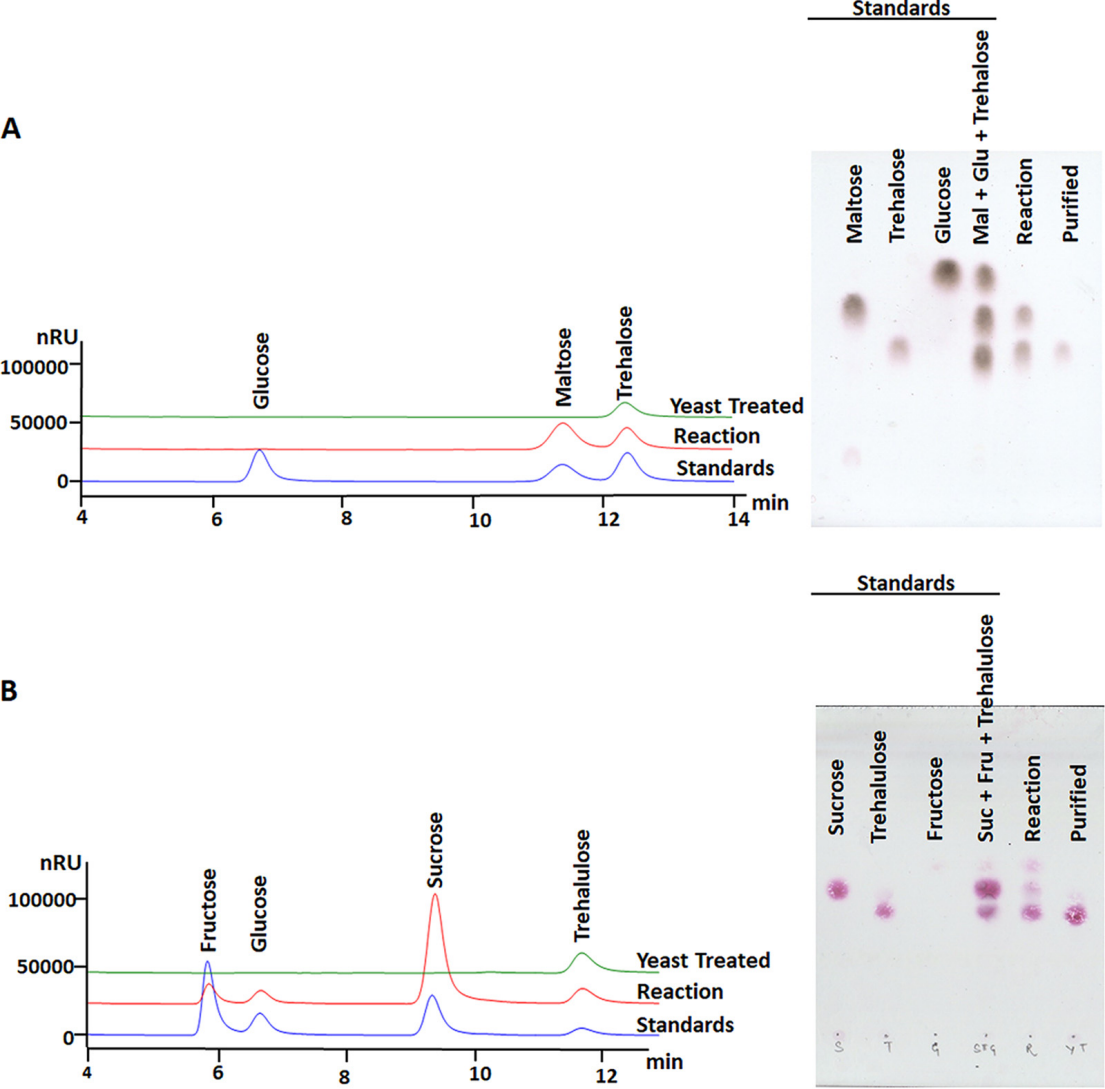
HPLC and TLC chromatograms showing sugar profiling before and after yeast treatment. The purified fractions of trehalose (A) and trehalulose (B) have been shown.

The enzymatically treated samples containing trehalose or trehalulose, were treated with baker’s yeast that consumed the fermentable sugars, e.g., glucose, fructose, maltose, and sucrose. The purity of trehalose and trehalulose was analyzed in TLC and HPLC (Fig. 4A and B).

## DISCUSSION

Trehalose synthase is a promising biocatalyst for executing a one-step reaction of trehalose production. With high substrate specificity and a simple maltose conversion reaction, trehalose synthase has extortionate application for trehalose production. However, its industrial use has been prompted to be limited by poor thermostability and low product yield (28). Apart from the reversible interconversion of maltose and trehalose, trehalose synthase may act on sucrose, catalyzing the transfer of glucosyl unit onto d-fructose, resulting in the biosynthesis of the sucrose isomer trehalulose. However, only a few trehalose synthases have been characterized so far with the dual catalytic activity of maltose and sucrose transformation into high-value rare sugar molecules (23, 25). This study identified a novel trehalose synthase gene (*treM*) from a thermal spring metagenome followed by gene cloning, heterologous expression, and its biochemical characterization for trehalose and trehalulose production. The sequence analysis of the *treM* revealed its inferior match with the genomic data in the public NCBI database. The results endorsed *treM* as a novel gene that might belong to an uncultured organism. The protein sequence-based phylogenetic analysis of TreM depicted its evolutionary closeness with Schlegelella thermodepolymerans, *Thermanaerothrix daxensis*, and *Anaerolineales bacterium*. Nevertheless, trehalose synthase has not been characterized from these organisms so far to the best of our knowledge.

The amino acid sequence alignment with previously characterized trehalose synthases, prediction of secondary structure, and creation of a 3D homology model of the protein identified the conserved A, B, and C domains, conserved motifs, and the critical catalytic residues in TreM, which were in agreement with the previous studies (19, 26). The SDS-PAGE analysis of recombinantly expressed and purified proteins determined the molecular mass of TreM (60 kDa) in the range of previously characterized trehalose synthases, e.g., Thermomonospora curvata (60 kDa) (23), Thermobifida fusca (66 kDa) (29), *Thermobaculum terrenum* (65 kDa) (19), Deinococcus geothermalis DSMZ 11300 (65 kDa) (27), *Pimelobacter* sp. R48 (62 kDa) (24), Deinococcus radiodurans R1 (64 kDa) (27), Picrophilus torridus (65 kDa) (30), Enterobacter hormaechei (65 kDa) (31), Deinococcus radiodurans ATCC 13939 (61 kDa) (27), and a saline-alkali soil-derived metagenomic enzyme (63 kDa) (32).

The biochemical enzyme characterization revealed that the neutral pH of the medium supports the maximum activity of TreM, which was similar to trehalose synthases from *E. hormaechei* (31) and T. thermophilus HB27 (20). However, a drastic decrease in the enzyme’s activity in the case of reactions performed in Tris buffer (pH 7.0) could be because of H-bond formation between the Tris molecule and the active site residues of trehalose synthase (33). This idea was in accordance with the previous reports on trehalose synthases from M. smegmatis (34), *P. torridus* (30), Arthrobacter aurescens (35), Corynebacterium glutamicum (33), and Pseudomonas sp. P8005 (36). A higher pH stability profile demonstrated by TreM at 5.0 to 9.0 pH was similar to the counterparts characterized from *P*. *torridus*, Pseudomonas sp. P8005, and Rhodococcus opacus (30, 36, 37). However, TreM was observed to have better stability at alkaline pH than that of Pseudomonas putida trehalose synthase, which showed a drastic decline in its residual activity at pH 8.6 (38).

Although, the metagenomic DNA fragment (*treM* gene) was obtained from a hyperthermophilic source, TreM showed the optimum catalytic activity at the mesophilic temperatures (45 to 50°C). The metagenomic DNA fragment encoding the TreM protein exhibits phylogenetic closeness with *Thermanaerothrix daxensis*, which grows between 50 to 73°C, with optimum growth at 65°C (39). At the nucleotide level, the metagenomic DNA fragment (*treM* gene) showed similarity with a genomic stretch of Schlegelella thermodepolymerans, which has an optimum growth temperature of 50 to 55°C (40). The high optimal growth temperature of an organism or the high temperature of the source of the metagenome does not imply that the enzymes encoded by the genomic sequences are adapted to work at high temperatures (41). Most of the intracellular enzymes of thermophilic bacteria may be found active at the organism’s growth temperature, whereas others may be functional at 10 to 20°C below the optimal growth temperature of the host (42). Furthermore, thermophilic enzymes that have been heterologously expressed in E. coli often exhibit different activity and stability profiles, compared with the same enzyme purified from the native organism, which could be due to differential folding pattern and bond formation, among others (42, 43).

**FIG 5:**
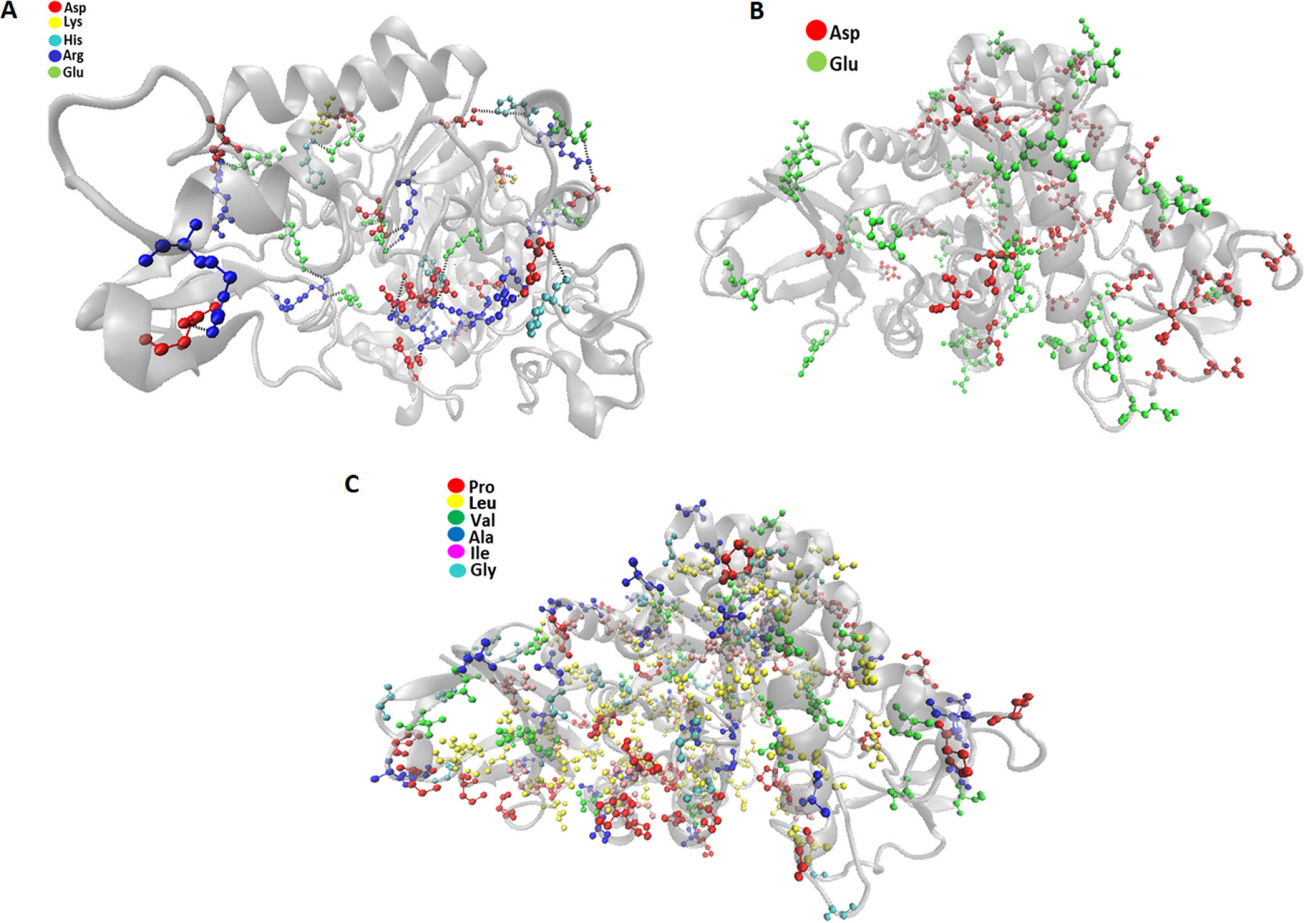
Distribution of Glu, Asp, Arg, and Lys residues, possibly involved in the formation of a salt bridge. A salt bridge has been represented between two ion pairs using a black dashed line. Distribution of acidic residues (A), Glu and Asp (B), and nonpolar residues (C) in TreM.

The temperature activity profile of TreM for trehalose synthesis was in accordance with the trehalose synthases characterized from *T. terrenum* (19), *D. geothermalis* (27), Meiothermus ruber (44), and *P. torridus* (30). A slight variation in the temperature optima of TreM for trehalose and trehalulose biosynthesis could be ascribed to the changes in the shape of the enzyme after binding with the different acceptor molecules (45). TreM discerned substantially high thermal stability at the optimal temperatures 45°C and 50°C. TreM exhibited a better heat tolerance than trehalose synthases characterized from C. glutamicum, *R. opacus*, *D. geothermalis*, Pseudomonas sp. P8005, *A. aurescens*, and D. radiodurans (Table S1). The high thermal stability of TreM could be ascribed to the presence 23 salt bridges, comprised of Glu (6.1%), Asp (6.8%), Arg (7.0%), and Lys (1.8%) residues (Fig. 5A and B). The presence of nonpolar residues, such as Leu (11.1%), Ala (7.4%), Pro (7.4%), Gly (6.27), Val (5.0%), and Ile (4.2%), in TreM (Fig. 5C) could enhance the structural stability of the protein. TreM has a higher percentage of nonpolar residues than its thermolabile counterparts (see Table S3 in the supplemental material). The nonpolar amino acids are often preferred in thermophilic proteins (46–48). The nonpolar residues are critical in creating hydrophobic interactions, such as van der Waals force and London dispersion force, stabilizing the folded configuration of proteins and increasing its structural stability (49). Many studies have endorsed the role of a high occurrence of Proline residues in according firmness to the protein (46, 50–52). At the same time, a reduced proportion of thermolabile amino acids, namely, Gln (4.4%), Asn (5%), Met (2.4%), and Cys (0.9%), further endorses the molecular aspect of TreM stability.

The activity of TreM was recorded to be unaffected by the introduction of various metals in the reaction medium, except Cu^2+^ that diminished its catalytic action. A similar metal activity profile was observed in the case of trehalose synthase from *E. hormaechei* (31). In the case of trehalose synthase from Pseudomonas putida, besides Cu^2+^, the presence of Zn^2+^ or Mn^2+^ severely affected the enzyme activity (38). Thermobifida fusca trehalose synthase was noted to be unaffected by the presence of Cu^2+^, Zn^2+^, and Mn^2+^ in the reaction (29). Tolerance to various metals is considered to be a favorable property of an enzyme for its use in diverse applications.

A slight decrease in the maltose-to-trehalose conversion during an extended period of catalysis at optimum temperature (Fig. 3A and S4A) could be due to a relatively higher rate of the reverse (trehalose to maltose) reaction. Similar results were presented in a trehalose synthase study of P. putida (38). The inclusion of the acceptor molecule in the reaction may boost the transglycosylation reaction of the GH13 enzyme (53). In contrast, the addition of acceptor molecules in the catalytic reaction of TreM was noted to be unfavorable, achieving nil gain in the product yield. This result might be due to the inhibition of trehalose synthase by the monosaccharide, glucose (29).

In the case of TreM, although the rate of trehalose production was noticed to be higher at the optimum reaction temperature (45°C), by-product formation increased with the reaction time course. The formation of by-products often limits the catalytic production of trehalose. Intriguingly, by-product formation declined with reducing the reaction temperature of TreM. This finding could be due to the higher accessibility of water molecules to the enzyme active site at the high temperature, resulting in the release of glucose rather than the formation of an α, α-1,1-glycosidic bond (54). Therefore, trehalose yield tends to be increased at lower temperatures (27, 30). The trehalose yield of about 74% achieved in the present study was comparable to that of trehalose synthases from Thermus antranikianii (76.8% at 40°C in 8 h) (55), *T. terrenum* (70% at 45°C in 10 h), *T. curvata* (70% at 60°C in 24 h), Pseudomonas sp. P8005 (70% at 37°C in 12 h) (36), *P. torridus* (71% at 20°C in 72 h) (30), and Pseudomonas stutzeri CJ (72% at 35°C in 19 h) (56) and the metagenomic trehalose synthase from saline-alkali soil (78% at 45°C in 18 h) (32).

The trehalulose yield of 90% (Fig. S4B) in a 24-h reaction was better than that in previous reports on trehalose synthase from *T. aquaticus* (81% in 96 h) (25) and *T. curvata* (80% in 24 h) (23). However, increasing the substrate (sucrose) concentration (300 mM to 1 M) negatively affected the catalytic conversion (Fig. S4B), which could be due to the increased viscosity in the reaction mixture, affecting the protein folding and kinetic performance of the enzyme (57). The utilization of low-cost feedstock (table sugar, muscovado, and sweet sorghum stalk juice) as glycosyl donors in the enzymatic reaction further validated the industrial importance of the novel trehalose synthase TreM, achieving a trehalulose yield comparable to that of sucrose.

In summary, the present investigation reports a novel trehalose synthase (TreM) identified from an extreme temperature thermal spring metagenome. It was found to be a thermostable enzyme that has a greater preference for industrial applications. The protein sequence and homology structure were analyzed to identify conserved motifs and critical catalytic residues. TreM was characterized for the production of trehalose and trehalulose from maltose and sucrose, respectively. Trehalose and trehalulose production were independent of the addition of acceptor molecules.

## MATERIALS AND METHODS

### Materials.

The standard sugars, namely, glucose, fructose, trehalose, and maltose monohydrate, were procured from Sigma-Aldrich (USA). Trehalulose was purchased from Carbosynth Ltd. (UK). Luria-Bertani broth, Luria-Bertani agar, yeast extract, peptone, malt extract, Coomassie blue, imidazole, Tris base, and sucrose were purchased from HiMedia (India). The restriction enzymes and molecular biology kits were procured from New England BioLabs (NEB; USA) and Thermo-Fischer Scientific (USA). The pJET1.2 cloning vector and DNA and protein ladders were obtained from Thermo-Fisher Scientific. The expression vector (pET28a) and host Escherichia coli BL21(DE3) were procured from Novagen. Ammonium persulphate, sodium acetate, sodium chloride, and isopropyl β-d-1-thiogalactopyranoside (IPTG) were purchased from Sigma-Aldrich. The nickel-chelating nitrilotriacetic acid (Ni-NTA) agarose gel was procured from Qiagen (Germany). Unrefined and partially refined sugars like muscovado, jaggery, and table sugar were procured from the local market. Sugarcane molasses was purchased from Saraswati Sugar Mills Ltd., Yamuna Nagar, Haryana, India. All the solvents for TLC and HPLC analyses were procured from CDH (India).

### Gene cloning and sequencing.

A putative trehalose synthase gene (*treM*) was amplified from the metagenomic DNA obtained from an extreme temperature (98°C) thermal spring, located in Tattapani, Chhattisgarh, India (58), by conducting PCR using sequence-specific primers and Phusion high-fidelity DNA polymerase (NEB, USA). The amplicon of the putative gene (*treM*) was cloned into the pJET1.2 vector and sequenced. The primers used for gene cloning and sequencing are listed in Table S2 in the supplemental material. Then, the putative gene (*treM*) was cloned in the expression vector pET28a, under the restriction sites NheI and XhoI. Subsequently, the *treM-p*ET28a construct was introduced into E. coli BL21(DE3) cells. As a control, the empty vector (pET28a) transformant was also generated.

### Sequence analysis.

The sequence of *treM* was compared with the corresponding sequences in the public NCBI nucleotide and protein databases by following the BLASTn and BLASTx algorithms. The domain analysis in the translated protein (TreM) was done using the Conserved Domain Database (CDD) tool. The secondary structure determination and preparation of the predicted 3D homology model of TreM were done by using the Phyre2 software (59). The homology model of TreM was superimposed over the best hit template (*Thermobaculum terrenum*) using Chimera 1.13 (https://www.cgl.ucsf.edu/chimera). The phylogenetic relation of TreM was determined by constructing a tree following the maximum likelihood method and the tensor train (TT) matrix-based model using Mega-X software version 10.2.1 (60, 61). The bootstrap consensus tree was worked out, taking 1,000 replicates. Salt bridges were predicted using the Visual Molecular Dynamics (VMD) software (62). The two oppositely charged atoms of each ion pair in salt bridges were determined by a distance cutoff of 3.2 Å. The hydrophobicity of the trehalose synthases was determined by the tool peptide 2.0 (http://peptide2.com/). The isoelectric points, net charge, and polar and nonpolar properties of the protein were determined by Emboss Pepstats (https://www.ebi.ac.uk/Tools/seqstats/emboss_pepstats/).

### Gene expression and protein purification.

E. coli transformants (pET28a-*treM*) were grown in Luria-Bertani (LB) broth medium, containing 0.5 mg·mL^−1^ kanamycin, at 37°C and 200 rpm. The protein production was induced in the transformants (optical density [OD], 0.5 to 06) by the addition of IPTG (0.3 mM) in the culture. The culture was incubated at 37°C and 150 rpm for 4 h. The cells were harvested by centrifugation at 6,000 rpm and 4°C for 10 min. The cells were suspended in 50 mM Tris buffer (pH 7.0) containing 50 mg·mL^−1^ lysozyme. After 30 min of incubation at room temperature, the cells were subjected to sonicating for 3 min (3-second pulse on, 15-second pulse off) at 4°C. The crude cell extract was obtained by centrifugation of cell debris at 10,000 rpm and 4°C for 20 min. The crude cell extract was passed through a 0.45-μm filter.

The His-tagged TreM protein was purified using nickel-chelating nitrilotriacetic acid (Ni-NTA) column chromatography. The column was equilibrated by using a binding buffer, comprised of 50 mM sodium phosphate buffer (pH 7.0), 20 mM imidazole, and 300 mM NaCl. The filtered crude cell extract was loaded onto the equilibrated column. Then, the column was washed with 50 mM sodium phosphate buffer (pH 7.0), containing 50 mM imidazole and 300 mM NaCl. The TreM protein was eluted in the elution buffer, comprised of 50 mM sodium phosphate buffer (pH 7.0), 200 mM imidazole, and 300 mM NaCl. The purified TreM fraction was dialyzed in a 14-kDa dialysis membrane to remove imidazole and NaCl from the protein preparation. TreM was concentrated in an Amicon Ultra-15 centrifugal filter with a 30-kDa cutoff. All steps of protein purification were performed at 4°C. To check the purity and molecular mass of TreM, the purified protein was loaded on 10% SDS-PAGE, and the gel was stained in Coomassie brilliant blue (Bio-Rad). Destaining of the gel was performed using a solution comprising of methanol: acetic acid: water in the ratio 4:1:5 to visualize the protein bands. The concentration of the protein was determined by Bradford’s method, taking bovine serum albumin as the standard.

### Enzyme activity assay.

The enzyme activity for trehalose synthesis was determined by conducting standard assays in 50 mM sodium phosphate buffer (pH 7.0), containing 100 mM maltose as the substrate and 0.1 mg·mL^−1^ TreM, and incubated at 45°C for 10 min. To determine the enzyme activity of TreM for trehalulose synthesis, 300 mM sucrose was treated with TreM (0.3 mg·mL^−1^) in sodium phosphate buffer (pH 7.0) at 50°C for 10 min. The reactions were stopped by boiling at 100°C for 10 min. The reaction products trehalose and trehalulose were analyzed and quantitated by analytical techniques.

### Qualitative and quantitative analysis of reaction products.

The reaction products trehalose and trehalulose were analyzed by thin-layer chromatography (TLC) and high-performance liquid chromatography (HPLC). HPLC, equipped with a 4.6 by 250-mm Zorbax-NH_2_ column, was operated with the column temperature 25°C, RID temperature of 50°C, and 1.4 mL min^−1^ flow rate of the mobile phase. A degassed solution of 75% acetonitrile and 25% Milli-Q water was used as the mobile phase.

The TLC was run in a glass chamber containing the mobile phase comprised of *N*-butanol, pyridine, and water in a 7:3:1 ratio for trehalose detection. The TLC was sprayed with 20% H_2_SO_4_ and then heated at 120°C for 10 min to visualize the sugar spots. In the case of trehalulose, TLC was run in the mobile phase, comprised of ethyl acetate, acetic acid, and water in a 3:3:1 ratio. Then, TLC was sprayed with 0.5% (wt/vol) 1-naphthyl ethylenediamine dihydrochloride and 5% sulfuric acid in methanol and charred at 104°C for 10 min until the spots appeared.

### Optimum pH and temperature.

Enzyme assays were conducted in the buffers of a 4.0 to 10.0 pH range. The buffers used were sodium acetate buffer (pH 4.0 and 5.0), sodium phosphate buffer (pH 6.0 to 8.0), and sodium bicarbonate buffer (pH 9.0 and 10.0). The reactions were performed with 100 mM maltose for trehalose biosynthesis, treated with 0.1 mg·mL^−1^ TreM at 45°C for 10 min. The reactions were performed using 300 mM sucrose, treated with 0.3 mg·mL^−1^ TreM at 50°C for 10 min for trehalulose biosynthesis. The reactions were stopped by denaturing the enzyme in a boiling water bath for 10 min.

The temperature profile of TreM was investigated by performing reaction assays in 50 mM sodium phosphate buffer (pH 7.0) in the temperature range of 10°C to 80°C. For trehalose biosynthetic activity, the reactions were conducted with 100 mM maltose and 0.1 mg·mL^−1^ TreM; whereas, for trehalulose synthesis, 300 mM sucrose was taken to treat with 0.3 mg·mL^−1^ TreM.

### Temperature and pH stability.

To determine the half-life of TreM, the enzyme aliquots, in 50 mM sodium phosphate buffer, were incubated at different temperatures (45°C, 50°C, and 60°C) for a long period of time (up to 5 days). The residual activity of the enzyme was measured at different time points by performing the standard enzyme assay. For pH stability, TreM was incubated in the buffers of 4.0 to 10.0 pH for 1 h at 45°C or 50°C followed by standard reaction assays for trehalose or trehalulose synthesis.

### Effect of metal ions.

The effect of different metal ions was examined on the activity of the trehalose synthase enzyme by performing reaction assays under optimal conditions for 1 h in the presence of different metal salts (1 mM), e.g., CaCl_2_, CuSO_4_, CoCl_2_, FeCl_3_, NaCl, KCl, LiCl, MnCl_2_, MgCl_2_, and ZnCl_2_, and EDTA. Boiling in a water bath for 10 min stopped the reactions. The reaction products were quantified using HPLC.

### TreM catalysis in variable substrate or enzyme concentrations.

For trehalose synthesis, 20 mM to 700 mM maltose was treated with 0.1 mg·mL^−1^ TreM for 72 h in 50 mM sodium phosphate buffer (pH 7.0) at 45°C. The trehalose produced was quantified by HPLC at different time points. For trehalulose synthesis, the enzyme assays were performed with 70 mM to 1 M sucrose, treated with 0.3 mg·mL^−1^ TreM in 50 mM sodium phosphate buffer (pH 7.0) at 50°C for 24 h. Trehalose yield was determined by high-performance liquid chromatography (HPLC) at different time points.

### Effect of acceptor molecule on TreM biocatalytic activity.

To examine the effect of the acceptor molecule on the trehalose or trehalulose catalysis, reaction assays were performed under optimal conditions for 1 h in the presence of different concentrations of glucose or fructose, respectively. The reaction products were quantified by HPLC.

### Purification of trehalose and trehalulose.

After completion of the enzymatic process, the samples were treated with Saccharomyces cerevisiae (baker’s yeast) for at 30°C for 24 h, with continuous shaking at 150 rpm. The yeast fermented the maltose, glucose, and fructose in the reaction mixture, leaving trehalose or trehalulose unutilized. The reaction products, before and after purification, were analyzed by TLC and HPLC.

### Statistical analysis.

All enzymatic reactions were performed in triplicates. The statistical difference between the treatments was determined by the analysis of variance test and Tukey’s *post hoc* test, using Minitab 20 statistical software.

### Data availability.

The sequence was submitted to NCBI under accession no. MZ293191.
