# Supplementary material for: A Novel Trehalose Synthase for the Production of Trehalose and Trehalulose
Source: Microbiol Spectr. 2021 Nov 24;9(3):e01333-21. doi: 10.1128/Spectrum.01333-21 (PMC8612140; doi:10.1128/Spectrum.01333-21)
Supplement: SUPPLEMENTAL FILE 1 — Supplemental material. Download SPECTRUM01333-21_Supp_1_seq7.pdf, PDF file, 1.5 MB [file spectrum01333-21_supp_1_seq7.pdf]

Table S1. Comparison of biochemical properties of trehalose synthases characterized from different sources.

Accession number	Organism	Mol wt. (kDa)	Optimum pH	Optimum temp (°C)	Trehalose Yield (%)	Trehalulose Yield (%)	Thermostability	Reference
CAF20645	*Corynebacterium glutamicum* (ATCC 13032)	70	7	35	69% (25°C) in 9h	-	80% (40 °C, 30 min)	1
WP_012853903	*Thermomonospora curvata* (DSM 43183)	60	6.5	35	70% (60°C) in 24h	80% (35°C) in 24h	NR	2
ZP_00057249	*Thermobifida fusca (*DSM 43792)	66	6.5	25	55-65% (25°C)	-	NR	3
AGF84773	*Rhodococcus opacus* (ATCC 41021)	79	7	25	67% (25°C)	-	100% (15–45 °C, 1 h) 0% (60 °C, 3h)	4
WP 028494267	*Thermus antranikianii*	112	7	60	76.8 %(40°C) in 8h; 62.6% (60°C) in 2h	-	NR	5
ACZ41252	*Thermobaculum terrenum*	65	7.5	45	70% (45 °C) in 10h	-	80% (70 °C, 30 min)	6
AEB92232	*Deinococcus geothermalis* (DSMZ 11300)	65	7.6	40	60.4% (40°C) in 24h	-	57% (55 °C, 8 h) 20% (60 °C, 2 h)	7
ACA35051	*Meiothermus ruber*	110	6.5	50	64% (20°C), 61% (30°C), 56% (40°C), 47% (50°C) in 24h	-	90% (60 °C, 5 h)	8
P72235.1	*Pimelobacter* sp. R48	62	7.5	20	81.8% at 5°C in 48h	-	NR	8
AEJ36289	*Deinococcus radiodurans* R1	64	7.6	30	58.2% (30 °C) in 24h	-	50% (40 °C, 28.5 h) (50 °C, 9.5 h) (55 °C, 30 min)	7
AFK94626	*Pseudomonas* sp. P8005	126	7.2	37	70% (37°C) in 12h	-	> 80% (10–40 °C, 30 min) 10% (50 °C, 30 min)	10
AAQ16097	*Thermus thermophilus* (ATCC 33923)	106	6.5	65	80% (65°C) in 48h	-	88% (65 °C, 30 min)	11
FJ545264	*Arthrobacter aurescens*	68	6.5	35	60% (35°C) in 8h	-	~ 100% (20–35 °C, 20 min) 10% (50 °C, 20 min)	12
WP_003897929.1	*Mycobacterium smegmatis*	68	7.2	37	42-45% (37°C) in 6h	-		13
AAT42654	*Picrophilus torridus* (DSM 9790)	65	6	45	71(20°C), 68 (30°C), 61 (45°C), 50 (60°C) in 72h	-	90% (60 °C, 20 min)	14

**ACI16355**	***Enterobacter hormaechei***	**65**	**6**	**37**	**48% (40°C) in 30 min**	**-**	**NR**	**15**
**AE000513**	***Deinococcus radiodurans* (ATCC 13939)**	**61**	**6.5**	**15**	**92% (5°C) in 48h**	**-**	**90% (40 °C, 30 min) 0% (60 °C, 2 h)**	**11**
**AAF26837**	***Pseudomonas stutzeri CJ 38***	**76**	**8.5**	**35**	**72% (35°C) in 19h**	**-**	**40% (55 °C, 1 h)**	**16**
**-**	***Thermus thermophilus* HB27**	**NR**	**9**	**50**	**38% (50°C) in 10h**	**-**	**NR**	**17**
**D86216**	***Thermus aquaticus (ATCC 33923)***	**105**	**6.5**	**65**	**80.7% (40 °C) in 48h**	**81% (40 °C) in 96 h**	**NR**	**18; US7927850B2**
**AY178981**	***Thermus caldophilus***	**110**	**6.3**	**40**	**86% (40°C)**	**-**	**NR**	**19, 20**
**NR**	**Metagenomics from saline-alkali soil**	**63**	**9**	**45**	**78% (45°C) in 18h**	**-**	**NR**	**21**
**MZ293191**	**Hot spring metagenome**	**59.6**	**7**	**45**	**66% (30 °C), 63% (45°C) in 3 h; 70% (20 °C), 74% (5°C) in 12h; 52% (45 °C) in 15 min**	**90% (50 °C) in 48 h**	**80% (45°C, 120 h) 60% (50°C, 120 h) 50% (60°C, 72 h)**	**This study**

Table S2. List of primers used for cloning and sequencing of *TreM*.

**Primers**	**5'-3' Primer Sequence**
**Cloning primer Forward (Gene F)**	GCTAGC ATGAGCGCATCCTACTGGTACAAAAA
**Cloning primer Reverse (Gene R)**	CTCGAG ACGCACTTCAATCCAGCGATATTGGTAGG
**Sequencing primer forward (Gene F)**	GCTAGC ATGAGCGCATCCTACTGGTACAAAAA
**Sequencing primer reverse (T7 reverse)**	TAGTTATTGCTCAGCGGTGG

Table S3. Percentage of non-polar residues in trehalose synthases as compared to TreM.

PROTEIN NAME	NON-POLAR (%)
**TreM**	**58.67**
Picrophilus torridus	**53.4**
Corynebacterium glutamicum	**52.9**
Rhodococcus opacus	**55.5**
Deinococcus radiodurans	**56.78**
Pseudomonas sp. P8005	**55.7**
Paenarthrobacter aurescens	**56.85**
Mycolicibacterium smegmatis	**55.987**
Thermobifida fusca	**55.9**
Enterobacter hormaechei	**56.93**
Thermobaculum terrenum	**54.8**

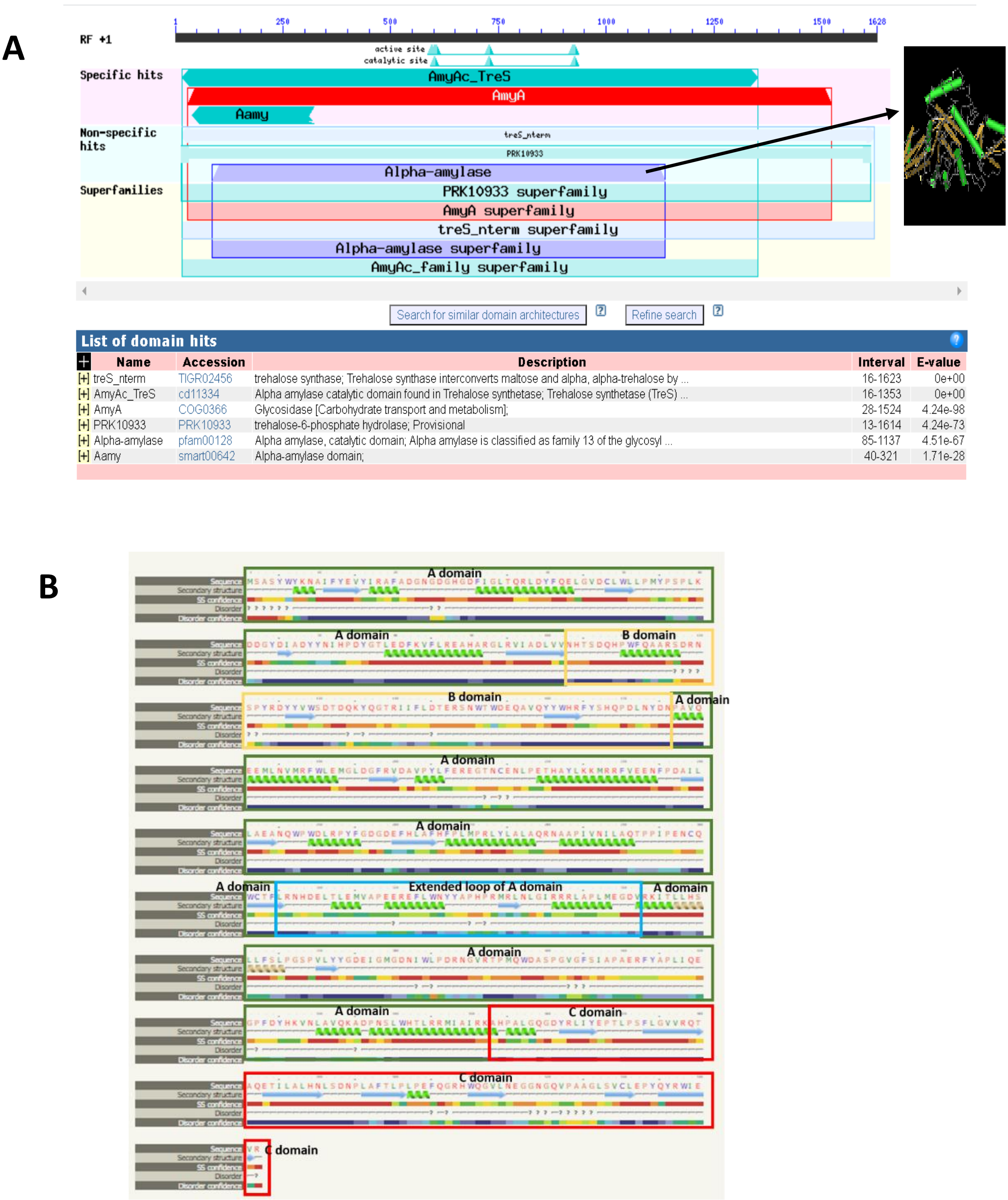

Figure S1. (A) Conserved domain analysis, showing an α-amylase domain and trehalose synthase domains in TreM. (B) Secondary structure analysis in TreM showing α-helix and β-sheets by using Phyre2 software. Three domains identified in the protein have been marked.

```
Pseudomonas_stutzeri          MSIPDNTYIEWLVSQSMLHAARERS---RHYAGQ----ARLWQRPYAQARP--RDASAIA    51
Enterobacter_hormaechei       -----------------------------------------------------------M    1
Deinococcus_radiodurans       -------------------------------------------------------MTQAH    5
Deinococcus_geothermalis      ------------------------------------------------------MTQTST    6
TreM                          ---------------------------------------------------------MSA    3
Thermus_antranikianii         ----------------------------------------------------------MD    2
Meiothermus_ruber             --------------------------------------------------------MGVD    4
Thermus_thermophilus          ----------------------------------------------------------MD    2
Thermus_caldophilus           ----------------------------------------------------------MD    2
Thermus_aquaticus             ----------------------------------------------------------MD    2
Arthrobacter_aurescens        ----------------MSFSP---------------QNPSQYF-TPKNTFELNAPGLQHD    28
Picrophilus_torridus          -------------------------------------------------------MLDNN    5
Pseudomonas_sp.               ----------------------------------------MYFQEFAMAKKPKAATFIKD    20
Thermobaculum_terrenum        -----------------------------------------------------MSNLNDD    7
Corynebacterium_glutamicum    --MTDTS--PLNSQPSADHHPDHAARPVLDAHGLIVEHESEEF-PVPAPAPGEQPWEKKN    55
Mycolicibacterium_smegmatis   ----------------M----EEHTQGSHVEAGIVEHPNAEDF-GH-------ARTLPTD    32
Rhodococcus_opacus            MPLEDSS--SAFADPSR----EGAAPPEHDLEGHIIEARSEDF-FH-------ARELQVD    46
Pimelobacter                  ----------------------------------------MSI-AE-------STVLGEE    12
Thermobifida_fusca            --MEKSM--TT-----------QPAPGARPT-----PTGSVPD-TF-------THAKPRD    32
Thermomonospora_curvata       ----------------------------MQM-----TGDPIPD-TF-------THEKPRD    19

Pseudomonas_stutzeri          SVWFTA------YPAAIITPEGGTVLEALGDDRLWSALSELGVQGIHNGPNKRSGGLRGR    105
Enterobacter_hormaechei       AGWHTRAIIYQIDTALFYDLNGDGCGDIAGITAKLRYIRRMGATVIWITP[illegible]YL-------    54
Deinococcus_radiodurans       PEWYKSAVFYELSVRTFQDGNGDGKGDFPGLTSRLDYLKNLGVDCLWLLPWFP-------    58
Deinococcus_geothermalis      SEWYKSAVFYELSVRTYADGNGDGKGDFPGLTGKLDYLKNLGVDCLWLLP[illegible]YP-------    59
TreM                          SYWYKNAIFYEVYIRAFADGNGDGHGDFIGLTQRLDYFQELGVDCLWLLPNYP-------    56
Thermus_antranikianii         PLWYKDAVIYQLHVRSFFDANDDGYGDFEGLRQKLPYLEALGVNTLWLMP[illegible]FQ-------    55
Meiothermus_ruber             PLWYKDAVIYQLHVRSFYDANDDGYGDFEGLRQKLPYLEALGVNTLWLMP[illegible]FQ-------    57
Thermus_thermophilus          PLWYKDAVIYQLHVRSFFDANNDGYGDFEGLRRKLPYLEELGVNILWLMP[illegible]FQ-------    55
Thermus_caldophilus           PLWYKDAVIYQLHVRSFFDANNDGYGDFEGLRRKLPYLEELGVNTLWLMP[illegible]FQ-------    55
Thermus_aquaticus             PLWYKDAVIYQLHVRSFFDANNDGYGDFEGLRRKLPYLEELGVNTLWLMP[illegible]FQ-------    55
Arthrobacter_aurescens        PHWYRKAVFYEVLVRAFADANGDGSGDFHGLIDKLDYLQWLGVDCLWLPP[illegible]FQ-------    81
Picrophilus_torridus          GLWYRDAVFYEVPVKSFYDSNNDGIGDFNGLTMKLDYLKKLGVDALWLLP[illegible]YK-------    58
Pseudomonas_sp.               PLWYKDAVIYQVHVKSYFDSNNDGIGDFAGLIAKLDYIADLGVNTIWLLP[illegible]YP-------    73
Thermobaculum_terrenum        PTWYKDAIIYEVGVRCFFDSNNDGSGDIPGLTAKLDYIESLGVTAIWLLP[illegible]YA-------    60
Corynebacterium_glutamicum    REWYKDAVFYEVLVRAFYDPEGNGVGSLKGLTEKLDYIQWLGVDCIWIPP[illegible]YD-------    108
Mycolicibacterium_smegmatis   TNWFKHAVFYEVLVRAFYDSNADGIGDLRGLTEKLDYIKWLGVDCLWLPP[illegible]YD-------    85
Rhodococcus_opacus            SEWFKTAVFYEVLVRAFFDSSGDGTGDLRGLTSKLDYLSWLGVDCLWLPP[illegible]YD-------    99
Pimelobacter                  PEWFRTAVFYEVLVRSFRDPNAGGTGDFRGLAEKLDYLQWLGVDCLWVPP[illegible]FS-------    65
Thermobifida_fusca            PYWYKHAVFYEVLVRGFYDSNGDGTGDLRGLIEKLDYLQWLGIDCLWLLP[illegible]YE-------    85
Thermomonospora_curvata       PYWYKHAVFYEVLVRGFSDSNDDGTGDLRGLINRLDYLQWLGIDCIWLLP[illegible]YQ-------    72
                                *.                . .   .  *       :  :*   :   *

Pseudomonas_stutzeri          EFTPTIDGNFDRI-SFDIDPSLGTEEQMLQLSRVAAAHNAIVIDDIVPAHTGKGADFRLA    164
Enterobacter_hormaechei       --TPFLDEGYDVSDHLQVDPRFGKLNDIIAFIEQARELGMQVIIELLIQHTSDAHPWFQQ    112
Deinococcus_radiodurans       --SPLRDDGYDVADYRGIHPDLGTLDDFKVFLREAHARGLRVIGDLVTNHTSSDHPWFQA    116
Deinococcus_geothermalis      --SPLRDDGYDVADYTDIHPDLGTLDDFKVFLREAHVRGLRVIGDLVTNHTSSDHPWFQA    117
TreM                          --SPLKDDGYDIADYYNIHPDYGTLEDFKVFLREAHARGLRVIADLVVNHTSDQHPWFQA    114
Thermus_antranikianii         --SPLRDDGYDISDYYQILPVHGSLEDFRRFLDEAHARGMRVIIELVLNHTSIDHPWFQE    113
Meiothermus_ruber             --SPLRDDGYDISDYYQILPVHGSLEDFKRFLDEAHEMGLRVIVELVLNHTSIDHPWFQE    115
Thermus_thermophilus          --SPLRDDGYDISDYYQILPVHGTLEDFKRFLDEAHGRGMKVIIELVLNHTSIDHPWFQE    113
Thermus_caldophilus           --SPLRDDGYDISDYYQILPVHGTLEDFKRLLDEAHGRGMKVIIELVLNHTSIDHPWFQE    113
Thermus_aquaticus             --SPLRDDGYDISDYYQILPVHGTLEDFT--VDEAHGRGMKVIIELVLNHTSIDHPWFQE    111
Arthrobacter_aurescens        --SPLRDGGYDIADYTSVLDEFGTISDFKRLVAEAHARGVRVIIDLPLNHTSDQHPWFQE    139
Picrophilus_torridus          --SPLKDDGYDISDYYSILPEYGTIDDFKNFIDTAHSMNIRVIADLVLNHVSDQHPWFIE    116
Pseudomonas_sp.               --SPRRDDGYDIAEYRGVHSDYGTMADAKRFIAQAHQRGLRVITELVINHTSDQHPWFQR    131
Thermobaculum_terrenum        --SPLKDGGYDISDYRSLHPDFGTIEDFKVFLDEAHRRGIRVITELVLNHTSDQHQWFRE    118
Corynebacterium_glutamicum    --SPLRDGGYDIRNFREILPEFGTVDDFVELVDHAHRRGLRVITDLVMNHTSDQHAWFQE    166
Mycolicibacterium_smegmatis   --SPLRDGGYDIRDFYKVLPEFGTVDDFVTLLDAAHRRGIRIITDLVMNHTSDQHEWFQE    143
Rhodococcus_opacus            --SPLRDGGYDIRDFRAVLPEFGTVDDFVQLFDQAHRRGIRVITDLVMNHTSDTHAWFQE    157
Pimelobacter                  --SPLRDGGYDVADYTGILPEIGTVEDFHAFLDGAHERGIRVIIDFVMNHTSDAHPWFQA    123
Thermobifida_fusca            --SPLRDGGYDVSDYMKILPEFGRISDFVELVEKAHQRGIRVITDLVMNHTSDQHPWFQA    143
Thermomonospora_curvata       --SPLRDGGYDISDYTKILPEFGDLGDFVELVDEAHRRGIRVIADLVMNHTSDQHPWFQA    130
                                :*  * .:*      :    *   :       *   .  :* ::   *..    :

Pseudomonas_stutzeri          EMA-Y--------GDYPGLYHMVEIREEDWELLPEVPAGRDSVNLLPPVVDRLKEKHYIV    215
Enterobacter_hormaechei       ARR-NPQ------SPYRDYYL-----------WSDTD-------------DD--------    133
Deinococcus_radiodurans       ARR-GPTLPDGSPNEYHDYYV-----------WSDEG-------------KEY-------    144
Deinococcus_geothermalis      ARR-GPTLPDGSPNEYFDYYV-----------WSDTG-------------TEY-------    145
TreM                          ARS-DRN------SPYRDYYV-----------WSDTD-------------QKY-------    136
Thermus_antranikianii         ARK--PG------SPMRDFYV-----------WSDTP-------------ERY-------    134
Meiothermus_ruber             ARK--PG------SPMRDWYV-----------WSDTP-------------EKY-------    136
Thermus_thermophilus          ARK--PG------SPMRDWYV-----------WSDTP-------------EKY-------    134
Thermus_caldophilus           ARK--PN------SPMRDWYV-----------WSDTP-------------EKY-------    134
Thermus_aquaticus             ARK--PN------SPMRDWYV-----------WSDTP-------------EKY-------    132
Arthrobacter_aurescens        SRK-DPT------GPYGDFYV-----------WSDTD-------------EKY-------    161
Picrophilus_torridus          SRS-SID------NPKRDWFI-----------WSDTP-------------EKF-------    138
Pseudomonas_sp.               ARKAKPG------SAARDFYV-----------WSDDD-------------QKY-------    154
Thermobaculum_terrenum        ARS-NPN------SPYRDYYV-----------WSDTD-------------DKY-------    140
Corynebacterium_glutamicum    SRR-DPT------GPYGDFYV-----------WSDDP-------------TLY-------    188
Mycolicibacterium_smegmatis   SRH-NPD------GPYGDFYV-----------WSDTS-------------DRY-------    165
Rhodococcus_opacus            SRA-DPD------GPYGDFYV-----------WSDRD-------------EGY-------    179
Pimelobacter                  SRS-DPD------GPYGDFYV-----------WSDTD-------------ELY-------    145
Thermobifida_fusca            SRH-DPD------GPYGNFYV-----------WSDTT-------------ERY-------    165
Thermomonospora_curvata       SRT-DPD------GPYGDFYM-----------WSDTD-------------DKY-------    152
                                           .   . :              :
```

```
Pseudomonas_stutzeri            GQLQRVIFFEPGIKDTDWSVTGEVTGVDGKVRRWVYLHYFKEGQPSLNWLDPTFAAQQLI    275
Enterobacter_hormaechei         --DTPPMFPGV--EKSIWTWD--------DEAGQYYRHMFYHHEPDLNLASPAVLKEVEN    181
Deinococcus_radiodurans         -ADTRIIFTDT--EVSNWTLD--------EQAGKYYWHRFFASQPDLNYDNPKVVEELHG    193
Deinococcus_geothermalis        -ADARIIFTDT--ETSNWTFD--------EMAGKYYWHRFFSSQPDLNYDNPRVQEELLN    194
TreM                            -QGTRIIFLDT--ERSNWTWD--------EQAVQYYWHRFYSHQPDLNYDNPAVQEEMLN    185
Thermus_antranikianii           -KGVRVIFQDF--EPSNWTFD--------PVAGAYYWHRFYHHQPDLNWDNPEVEKAMHQ    183
Meiothermus_ruber               -KGVRVIFQDF--ETSNWTFD--------PVAGAYYWHRFYHHQPDLNWDNPEVEKAMQE    185
Thermus_thermophilus            -KGVRVIFKDF--ETSNWTFD--------PVAKAYYWHRFYWHQPDLNWDNPEVEKAIHQ    183
Thermus_caldophilus             -KGVRVIFKDF--ETSNWTFD--------PVAKAYYWHRFYWHQPDLNWDNPEVEKAIHQ    183
Thermus_aquaticus               -KGVRVIFKDF--ETSNWTFD--------PVAKAYYWHRFYWHQPDLNWDSPEVEKAIHQ    181
Arthrobacter_aurescens          -QDARIIFVDT--EESNWTFD--------PIRRQFFWHRFFSHQPDLNFENPKVIEALYD    210
Picrophilus_torridus            -KEARIIFIDT--EKSNWTYD--------PETKQYYFHRFYSSQPDLNYDNPDVRNEVKK    187
Pseudomonas_sp.                 -DGTRIIFLDT--EKSNWTWD--------PVAGQYFWHRFYSHQPDLNFDNPQVMKAVLS    203
Thermobaculum_terrenum          -KDARIIFIDT--ERSNWTWD--------QEAGKYYWHRFYSHQPDLNYDNPKVQQEILD    189
Corynebacterium_glutamicum      -NEARIIFVDT--EESNWTYD--------PVRGQYFWHRFFSHQPDLNYDNPAVQEAMLD    237
Mycolicibacterium_smegmatis     -PDARIIFVDT--EESNWTFD--------PVRRQFYWHRFFSHQPDLNYDNPAVQEAMLD    214
Rhodococcus_opacus              -PEARIIFVDT--ETSNWTWD--------PVRKQYYWHRFFSHQPDLNYDNPDVQDAMID    228
Pimelobacter                    -QDARVIFVDT--EPSNWTWD--------QTRGQYYWHRFFHHQPDLNFDNPKVQDAMLE    194
Thermobifida_fusca              -SDARIIFIDT--EQSNWTYD--------EVRGQYYWHRFFSHQPDLNFENPDVQDAILE    214
Thermomonospora_curvata         -PDARIIFVDT--EVSNWTYD--------PVRGQYYWHRFFSHQPDLNYDNPAVQEAMLE    201
                                       :*      : : *:               : * *    :*.**  .* .

Pseudomonas_stutzeri            IGDALHAIDVTGARVLRLDANGFLGVERRAEGTAWSEGHPLSVTGNQLLAGAIRKAGGFS    335
Enterobacter_hormaechei         IIIFWLK---LGVSGFRLDAASHLTKQAGRGDEKR--GLWILEHLRCLIGQ-RN-PDAIL    234
Deinococcus_radiodurans         AARFWLD---LGLDGFRVDAVPYLIEREGTSCENLPETHEILKGFRAMVDR-EY-PGRLL    248
Deinococcus_geothermalis        VLRFWLD---LGLDGFRVDAVPYLIEREGTNCENLPETHAILQKLRRVVDE-EY-PGRLL    249
TreM                            VMRFWLE---MGLDGFRVDAVPYLFEREGTNCENLPETHAYLKKMRRFVEE-NF-PDAIL    240
Thermus_antranikianii           VMFFWAD---MGVDGFRLDAIPYLYEREGTNCENLPETIEAVKRLRKALEE-RYGPGKVL    239
Meiothermus_ruber               VMFFWAD---LGVDGFRLDAIPYLYEREGTSCENLPETIAAVKRLRAALEK-RYGPGKIL    241
Thermus_thermophilus            VMFFWAD---LGVDGFRLDAIPYLYEREGTSCENLPETIEAVKRLRKALEE-RYGPGKIL    239
Thermus_caldophilus             VMFFWAD---LGVDGFRLDAIPYLYEREGTSCENLPETIEAVKRLRKALEE-RYGPGKIL    239
Thermus_aquaticus               VMFFWAD---LGVDGFRLDAIPYLYEREGTSCENLPETHEFLKRLRKALEE-RYGPGKIL    237
Arthrobacter_aurescens          VVRFWLD---QGIDGFRADAIPYLFEEDGTNCENLPATHTFLQDLRRMVDS-NY-PGRVI    265
Picrophilus_torridus            VIRYWLD---LGLDGFRADAVPYLFKRENTNCENLPETHNFFKEIRKMMDE-DY-PGTIL    242
Pseudomonas_sp.                 VMRYWLD---LGIDGLRLDAIPYLIERDGTNNENLAETHNVLKQIRAEIDA-HY-PDRML    258
Thermobaculum_terrenum          IVGYWLD---MGVDGLRLDAVPYLYEREGTNCENLPETHEFLKKLRKFVDD-NW-PNRML    244
Corynebacterium_glutamicum      VLRFWLD---LGLDGFRLDAVPYLFEREGTNGENLKETHDFLKLCRSVIEK-EY-PGRIL    292
Mycolicibacterium_smegmatis     VLRFWLD---LGIDGFRLDAVPYLFEREGTNCENLPETHAFLKRCRKAIDD-EY-PGRVL    269
Rhodococcus_opacus              VLRFWLD---LGIDGFRLDAVPYLFEREGTNCENLPETHAFLKKCRAVIDA-EY-PGRAL    283
Pimelobacter                    AMAFWLD---MGLDGFRLDAVPYLYERPGTNGENLPETHEMLKRVRRFVDD-NY-PDRVL    249
Thermobifida_fusca              VMRFWLD---LGIDGFRLDAVPYLYEREGTNCENLKETHEFLKRIRAEVDR-LY-PDRVL    269
Thermomonospora_curvata         VLRFWLD---LGIDGFRLDAVPYLAREGTNCENLPETHAYLKRVRAEVDR-LY-PDRVL    256
                                           *    :* **   .*  .                .  :      .

Pseudomonas_stutzeri            FQELNLTIDDIA-----------AMSHGGADLSYDFITRPAYHHALLTGDTEFLRMMLRE    384
Enterobacter_hormaechei         LGEVDVEVEAYKDYFGQ---------NDRLNLVLNFWLNKYFYVSLAEKSARPLRNAVKK    285
Deinococcus_radiodurans         LAEANQWPEEVVEYFGTEA-------EPEFHMCFNFPVMPRLYMSLKREDTSSIREIMGR    301
Deinococcus_geothermalis        LAEANQWPEEVVEYFGTET-------HPEFHMCFNFPVMPRLFMSLKREDTTSIREIMAR    302
TreM                            LAEANQWPWDLRPYFGD---------GDEFHLAFHFPLMPRLYLALAQRNAAPIVNILAQ    291
Thermus_antranikianii           LAEANMWPEETLPYFGE---------GDGVHMAYNFPLMPRLFLALRREDRGPIEAMLQE    290
Meiothermus_ruber               LAEANMWPEETLPYFGE---------GDGVHMAYNFPLMPRIFMALRREDRRPIEAMLQE    292
Thermus_thermophilus            LAEANMWPEETLPYFGD---------GDGVHMAYNFPLMPRIFMALRREDRGPIETMLKE    290
Thermus_caldophilus             LAEANMWPEETLPYFGD---------GDGVHMAYNFPLMPRIFMALRREDRGPIETMLKE    290
Thermus_aquaticus               LAEVNMWPEETLPYFGD---------GDGVHMAYNFPLMPRIFMALRREDRGPIETMLKE    288
Arthrobacter_aurescens          IAEANQPPHEVVEYFGTEE-------APECHMAFHFPIMPRLYYALRDQKAAPIIETLRN    318
Picrophilus_torridus            LAEANQWPTETKAYFGN---------GDEFHMAFNFPLMPRIFIALARSDYYPIMDIIKQ    293
Pseudomonas_sp.                 LAEANQWPEDTQLYFGDRKGD----DGDECHMAFHFPLMPRMYMALAQEDRFPITDILRQ    314
Thermobaculum_terrenum          LAEANQWPEDVVAYFGN---------GDECHMAYHFPIMPRMYMALRREDRHPITEILRR    295
Corynebacterium_glutamicum      LAEANQWPQDVVEYFGEKDK------GDECHMAFHFPLMPRIFMGVRQGSRTPISEILAN    346
Mycolicibacterium_smegmatis     LAEANQWPADVVAYFGDPDT-----GGDECHMAFHFPLMPRIFMAVRRESRFPISEILAQ    324
Rhodococcus_opacus              LAEANQWPSDVVEYFGEPDV------GDECHMAFHFPLMPRIFMAVRRQNRFPISEILAQ    337
Pimelobacter                    LYEANQWPTDVVEYFGPEEREDGTVVGPESHMAFHFPVMPRIFMAVRRESRFPISEIMEQ    309
Thermobifida_fusca              LSEANQWPADVVDYFGDYES-----GGDECHMNFHFPLMPRMFMAVRREQRYPISEILAQ    324
Thermomonospora_curvata         LAEANQWPADVVEYFGDPAT-----GGDECHMAFHFPVMPRIFMAVRREQRYPISEIMAQ    311
                                : * :                          .:   .*        . .:    .    :    : .

Pseudomonas_stutzeri            VHAFGIDPASLIHALQNHDELTLELVHFWTLHAYDHYHYKGQTLPGGHLREHIREEMYER    444
Enterobacter_hormaechei         MIVP-PDSCCFANWLRNHDELDLEGIGKKA-----------------------KQTVIDT    321
Deinococcus_radiodurans         LPKI-PSFGQWCTFLRNHDELTLEMVTDDE-----------------------RAFMYAA    337
Deinococcus_geothermalis        LPKL-PSFGQWATFLRNHDELTLEMVTEDE-----------------------RAFMYAA    338
TreM                            TPPI-PENCQWCTFLRNHDELTLEMVAPEE-----------------------REFLWNY    327
Thermus_antranikianii           TEGI-PESAQWALFLRNHDELTLEKVTEEE-----------------------REFLWEV    326
Meiothermus_ruber               TEGI-PESAQWALFLRNHDELTLEKVTEEE-----------------------REFLWEI    328
Thermus_thermophilus            TEGI-PETAQWALFLRNHDELTLEKVTEEE-----------------------REFMYEA    326
Thermus_caldophilus             TEGI-PETAQWALFLRNHDELTLEKVTEEE-----------------------REFMYEA    326
Thermus_aquaticus               AEGI-PETAQWALFLRNHDELTLEKVTEEE-----------------------REFMYEA    324
Arthrobacter_aurescens          TPEI-PKGAQWGTFLRNHDELTLEMVPAEE-----------------------RAAMLGW    354
Picrophilus_torridus            TLPI-PDNCDWCIFLRNHDELTLEMVTDSE-----------------------RDIMYRE    329
Pseudomonas_sp.                 TPEI-PANCQWAIFLRNHDELTLEMVTDKE-----------------------RDYLWNY    350
Thermobaculum_terrenum          TPPI-PETCQWALFLRNHDELTLEMVTDEE-----------------------RDYMYHE    331
Corynebacterium_glutamicum      TPEI-PKTAQWGIFLRNHDELTLEMVSDEE-----------------------RSYMYSQ    382
Mycolicibacterium_smegmatis     TPPI-PDTAQWGIFLRNHDELTLEMVTDEE-----------------------RDYMYAE    360
Rhodococcus_opacus              TPPI-PSSCQWGIFLRNHDELTLEMVSDEE-----------------------RDYMYSE    373
Pimelobacter                    TPAI-PEGCQWGIFLRNHDELTLEMVTDED-----------------------RDYMWGE    345
Thermobifida_fusca              TPPI-PRNCQWAIFLRNHDELTLEMVSDEE-----------------------RDYMYSE    360
Thermomonospora_curvata         TPKI-PENCQWGIFLRNHDELTLEMVTDEE-----------------------RDYMYAE    347
                                               *:***** ** :                                :  :
```

```
Pseudomonas_stutzeri            LTGEHAPYNLKFVTNGVSCTTASVIAAALNIRDLDAIGPAEVEQIQRLHILLVMFNAMQP     504
Enterobacter_hormaechei         FAPDEE---MSVYQRGIR-------------RRLAPMLNGDRKRLAFCHAVLFSL----P     361
Deinococcus_radiodurans         YAPDAR---MKI-NVGIR-------------RRLAPLLDNDRRRIELLNTVLLAL----P     376
Deinococcus_geothermalis        YAPDAR---MKI-NVGIR-------------RRLAPLLDNDRRRIELLTTVLLAL----P     377
TreM                            YAPHPR---MRL-NLGIR-------------RRLAPLMEGDVRKITLLHSLLFSL----P     366
Thermus_antranikianii           YAPDPR---YRI-NLGIR-------------RRLMPLLGGDRRRFELLHALLFTL----K     365
Meiothermus_ruber               YAPDPR---FRI-NLGIR-------------RRLMPLLGGDRRRYELLQALLLTL----K     367
Thermus_thermophilus            YAPDPK---FRI-NLGIR-------------RRLMPLLGGDRRRYELLTALLLTL----K     365
Thermus_caldophilus             YAPDPK---FRI-NLGIR-------------RRLMPLLGGDRRRYELLTALLLTL----K     365
Thermus_aquaticus               YAPDPK---FRI-NLGIR-------------RRLMPLLGGDRRRYELLTALLLTL----K     363
Arthrobacter_aurescens          YAPDPR---MRA-NIGIR-------------RRLAPLLDNSRSEIELINALLLSL----P     393
Picrophilus_torridus            YAKIPK---MRL-NLGIR-------------RRLAPLADNDINTIELLNALIFSL----P     368
Pseudomonas_sp.                 YAADRR---ARI-NLGIR-------------RRLAPLVERDRRRVELLNSLLLSM----P     389
Thermobaculum_terrenum          YAKDPR---MRL-NLGIR-------------RRLAPLLDNSERRIQLMHLLLFTL----P     370
Corynebacterium_glutamicum      FASEPR---MRA-NVGIR-------------RRLSPLLEGDRNQLELLHGLLLSL----P     421
Mycolicibacterium_smegmatis     YAKDPR---MKA-NVGIR-------------RRLAPLLENDRNQIELFTALLLSL----P     399
Rhodococcus_opacus              YAQDPR---MKA-NIGIR-------------RRLAPLLENDRNQLELFTALLLSL----P     412
Pimelobacter                    YAKDPR---MKA-NIGIR-------------RRLAPLLDNDTNQIELFTALLLSL----P     384
Thermobifida_fusca              YAKDPR---MRA-NMGIR-------------RRLAPLLENDLNQIKLFTALLLSL----P     399
Thermomonospora_curvata         YAKDPR---MKA-NIGIR-------------RRLAPLLDNDRNQLELFTALLLSL----P     386
                                 :             *:               * *  :   .         ::. :

Pseudomonas_stutzeri            GVFALSGWDLVGALPLAPEQVEHLMGDGDTRWINR---------------GGYDLADLAP     549
Enterobacter_hormaechei         GVPVMR--------------YGDEIGMGDDLDLEERYAVRTPMQWAGSQGGGFSDAD--P     405
Deinococcus_radiodurans         GSPILY--------------YGDEIGMGDDLGLPDRNGVRTPMQWNAGTSGGFSTAQ--P     420
Deinococcus_geothermalis        GSPILY--------------YGDEIGMGDNLSLADRNGVRTPMQWNAGISGGFSTAL--P     421
TreM                            GSPVLY--------------YGDEIGMGDNIWLPDRNGVRTPMQWDASPGVGFSIAP--A     410
Thermus_antranikianii           GSPILY--------------YGDEIGMGDNPTLGDRNGVRTPMQWSADRNAGFSRAP--Y     409
Meiothermus_ruber               GSPILY--------------YGDEIGMGDNPTLGDRNGVRTPMQWYADRNAGFSRAP--Y     411
Thermus_thermophilus            GTPIVY--------------YGDEIGMGDNPTLGDRNGVRTPMQWSQDRNAGFSRAP--Y     409
Thermus_caldophilus             GTPIVY--------------YGDEIGMGDNPTLGDRNGVRTPMQWSQDRNAGFSRAP--Y     409
Thermus_aquaticus               GTPIVY--------------YGDEIGMGDNPTLGDRNGVRTPMQWSQDRIVAFSRAP--Y     407
Arthrobacter_aurescens          GSPFLY--------------YGDEIGMGDNIWLDDRDAVRTPMQWNPDRNAGFSSAD--P     437
Picrophilus_torridus            GTPIIY--------------YGDEIGMGDNIVLGDRNGVRTPMQWSYDRNAGFSMAD--S     412
Pseudomonas_sp.                 GTPTLY--------------YGDEIGMGDNIVLGDRDGVRTPMQWSIDRNGGFSRAD--P     433
Thermobaculum_terrenum          GTPIIY--------------YGDEIGMGDNVVLGDRDGVRTPMQWSGDRNAGFSRAN--P     414
Corynebacterium_glutamicum      GSPVLY--------------YGDEIGMGDNIWLHDRDGVRTPMQWSNDRNGGFSKAD--P     465
Mycolicibacterium_smegmatis     GSPVLY--------------YGDEIGMGDIIWLGDRDSVRTPMQWTPDRNAGFSKAT--P     443
Rhodococcus_opacus              GSPVLY--------------YGDEIGMGDNIWLGDRDSVRTPMQWTPDRNAGFSRAD--P     456
Pimelobacter                    GSPVLY--------------YGDEIGMGDNIWLGDRDGVRTPMQRTPDRNVGFSAAT--P     428
Thermobifida_fusca              GSPVLY--------------YGDEIGMGDNIWLGDRDSVRTPMQWTPDRNAGFSRCD--P     443
Thermomonospora_curvata         GSPVLY--------------YGDEIGMGDNIWLGDRDSVRTPMQWTPDRNAGFSRCD--P     430
                                *   :                 . :* **   :                  .:. .
```

Figure S2. Multiple sequence alignment among the previously characterized trehalose synthases and TreM. The blue boxes indicate conserved motifs. Red arrows indicate residues critical in substrate binding and catalytic function.

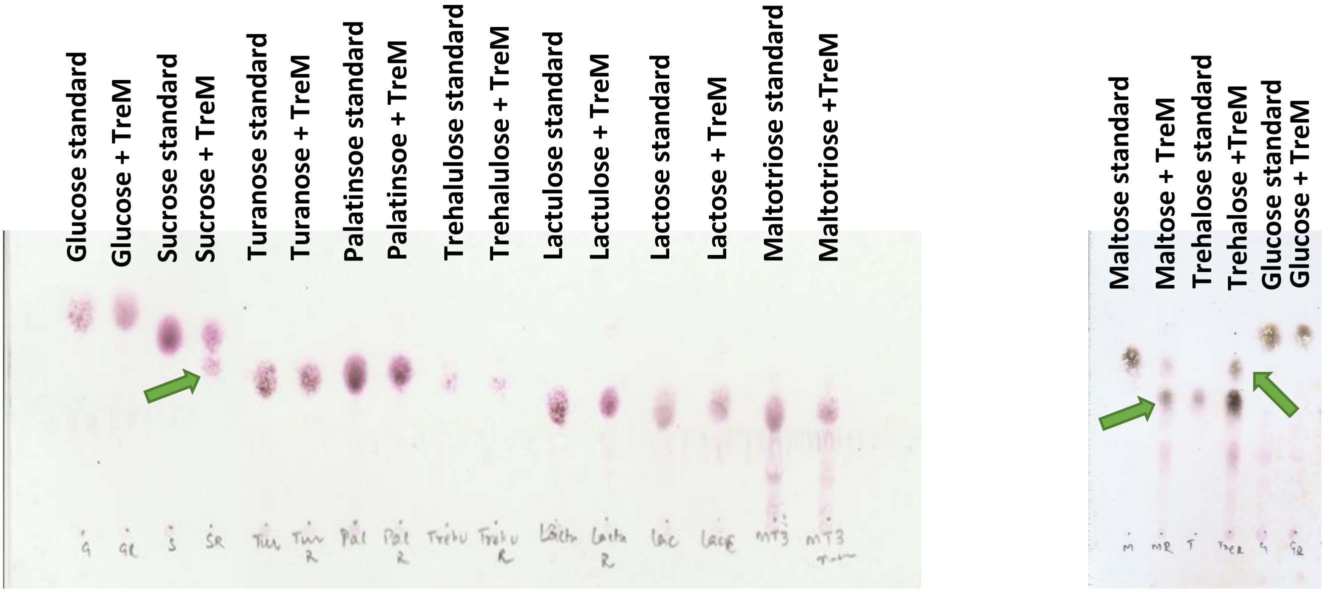

Figure S3. TLC showing catalytic reaction of TreM towards different substrates. Various substrates (5%) viz. maltotriose, turanose, trehalulose, palatinose, trehalose, lactose, glucose, sucrose, maltose, and lactulose, were treated with 0.3 mg mL^{-1} TreM in 50 mM sodium phosphate buffer (pH 7.0), at 45 °C for 24 h. The reactions were stopped by boiling the reaction mixtures in hot water bath for 10 min. TreM showed catalytic activity with the substrates: sucrose, maltose, and trehalose (as shown in the figures by green tags).

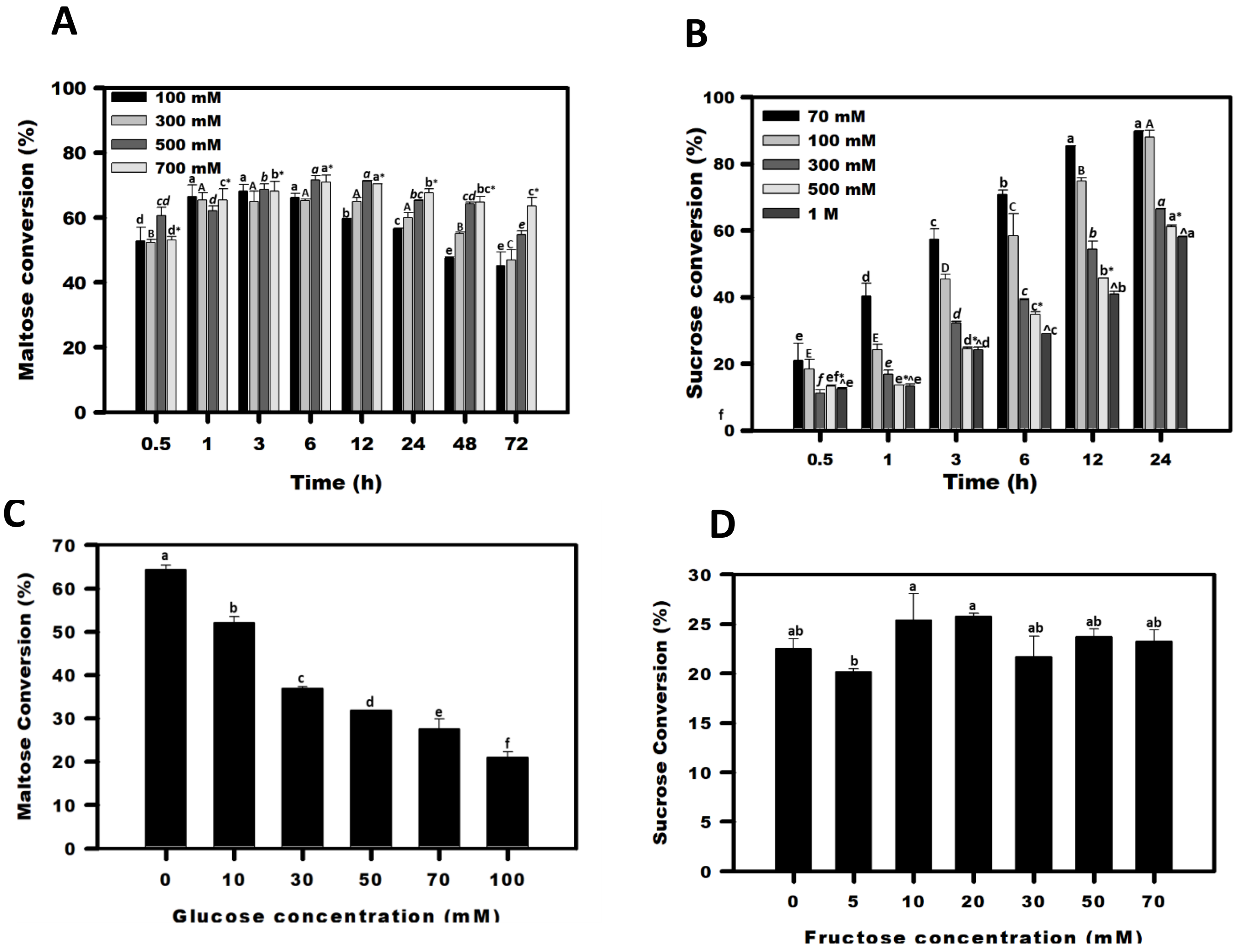

Figure S4. Effect of substrate concentration on the yield of (A) trehalose and (B) trehalulose, with respect to time. Effect of acceptor molecules, (C) Glucose and (D) fructose, on trehalose and trehalulose biosynthesis, respectively. Mean values not sharing common alphabets in the bars of same pattern are statistically different at $p < 0.05$.

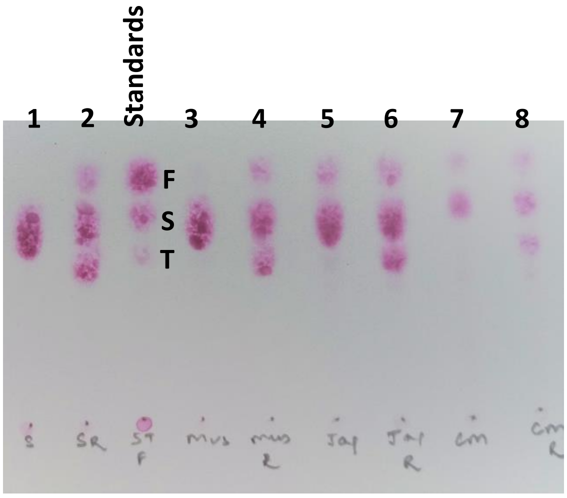

Lane1: Refined sugar control

Lane 2: Refined sugar reaction

Lane 3: Muscovado control

Lane 4: Muscovado reaction

Lane 5: Jaggery control

Lane 6: Jaggery reaction

Lane 7: Cane molasses control

Lane 8: Cane molasses reaction

Figure S5. Trehalulose biosynthesis from low-cost feedstocks. The low-cost feedstocks viz. cane molasses, muscovado, jaggery, and table sugar was treated with TreM (0.3 mg mL^{-1}) in 50 mM sodium phosphate buffer (pH 7.0) at 50°C, for 12 h. The reactions were stopped denaturing the enzyme in a boiling water bath for 10 min. The reaction product, trehalulose, was analyzed using TLC. F: fructose, S: sucrose, T: Trehalulose

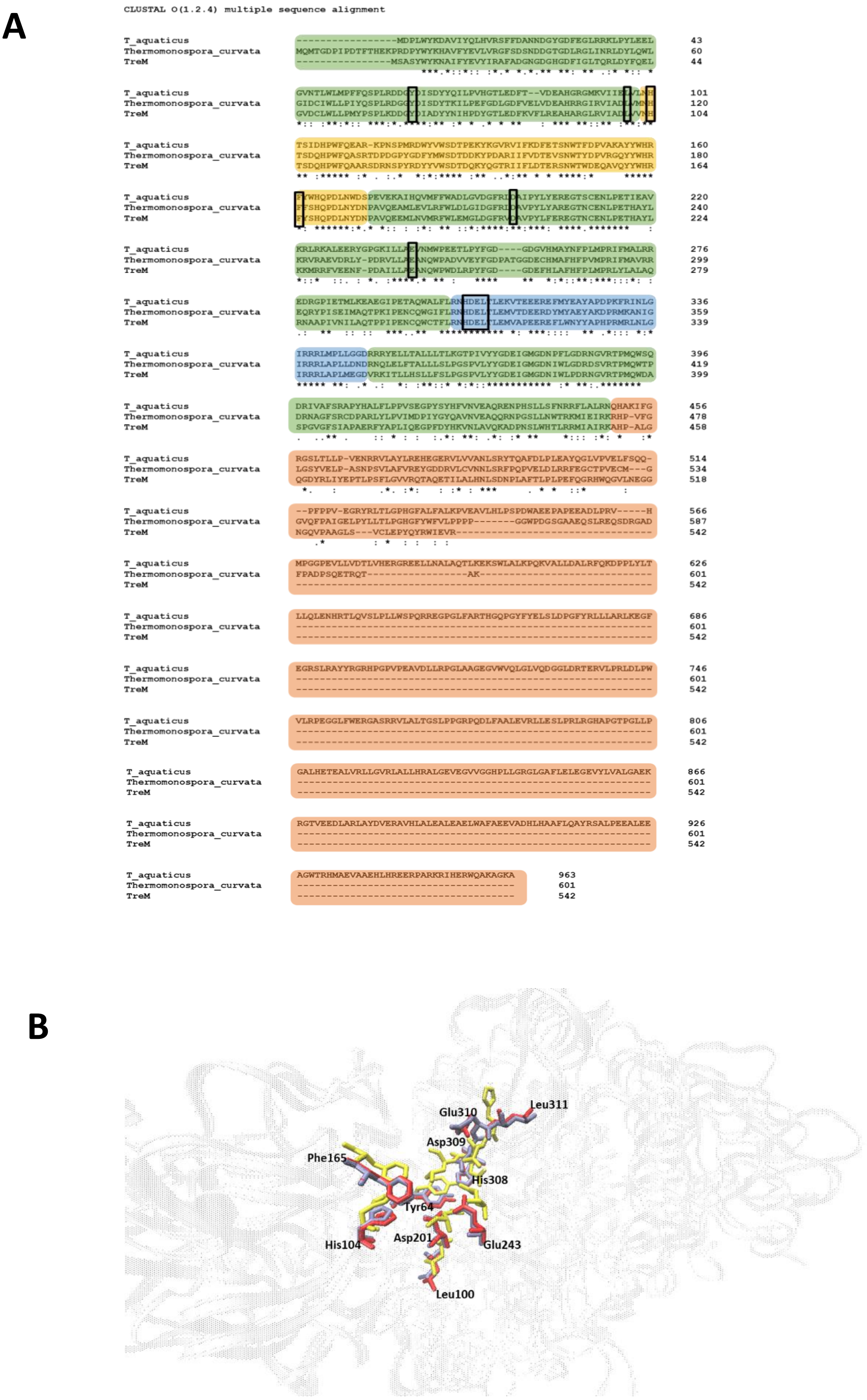

Figure S6. (A) Multiple sequence alignment among trehalose synthases from *T. curvata*, *T. aquaticus*, and TreM. The blue boxes indicate conserved residues responsible for enzymatic activity, substrate recognition, and product formation. (B) superimposition of 3-D model of *T. curvata*, *T. aquaticus* over TreM. The conserved catalytic residues are marked in the model.

References:

1. Kim TK, Jang JH, Cho HY, Lee HS, Kim YW. 2010. Gene cloning and characterization of a trehalose synthase from *Corynebacterium glutamicum* ATCC13032. *Food Sci Biotechnol* 19: 565-569.
2. Liang J, Huang R, Huang Y, Wang X, Du L, Wei, Y. 2013.Cloning, expression, properties, and functional amino acid residues of new trehalose synthase from *Thermomonospora curvata* DSM 43183. *J Mol Catal B Enzym* 90:26-32
3. Wei YT, Zhu QX, Luo ZF, Lu FS, Chen FZ, Wang QY, Huang RB. 2004. Cloning, expression and identification of a new trehalose synthase gene from *Thermobifida fusca* genome. *Acta Biochim Biophys Sin* 36: 477-484.
4. Yan J, Qiao Y, Hu J, Ding H. 2013. Cloning, expression and characterization of a trehalose synthase gene from *Rhodococcus opacus*. *Protein J* 32: 223-229.
5. Lin YF, Su PC, Chen PT. 2020. Production and characterization of a recombinant thermophilic trehalose synthase from *Thermus antranikianii*. *J Biosci Bioeng* 129: 418-422.
6. Wang J, Ren X, Wang R, Su J, Wang F. 2017. Structural characteristics and function of a new kind of thermostable trehalose synthase from *Thermobaculum terrenum*. *J Agr Food Chem* 65*:* 7726-7735.
7. Filipkowski P, Panek A, Agnieszka F, Pietrow O, Synowiecki J. 2012. Expression of *Deinococcus geothermalis* trehalose synthase gene in *Escherichia coli* and its enzymatic properties. *Afr J Biotechnol* 11:13131-13139
8. Zhu Y, Wei D, Zhang J, Wang Y, Xu H, Xing L, Li M. 2010. Overexpression and characterization of a thermostable trehalose synthase from *Meiothermus ruber*. *Extremophiles 14:* 1-8.
9. Nishimoto T, Nakano M, Nakada T, Chaen H, Fukuda S, Sugimoto T, Tsujisaka Y. 1996. Purification and properties of a novel enzyme, trehalose synthase, from *Pimelobacter* sp. R48. *Biosci Biotechnol Biochem* 60: 640-644.
10. Gao Y, Xi Y, Lu XL, Zheng H, Hu B, Liu XY, Jiao BH. 2013. Cloning, expression and functional characterization of a novel trehalose synthase from marine *Pseudomona*s sp. P8005. *World J Microbiol Biotechnol* 29: 2195-2206.

11. Wang JH, Tsai MY, Chen JJ, Lee GC, Shaw JF. 2007. Role of the C-terminal domain of *Thermus thermophilus* trehalose synthase in the thermophilicity, thermostability, and efficient production of trehalose. *J Agric Food Chem* 55: 3435-3443.
12. Xiuli W, Hongbiao D, Ming Y, Yu Q. 2009. Gene cloning, expression, and characterization of a novel trehalose synthase from *Arthrobacter aurescens*. *Appl Microbiol Biotechnol* 83: 477-482.
13. Pan YT, Koroth Edavana V, Jourdian WJ, Edmondson R, Carroll JD, Pastuszak I, Elbein AD. 2004. Trehalose synthase of *Mycobacterium smegmatis*: purification, cloning, expression, and properties of the enzyme. *Eur J Biochem* 271: 4259-4269.
14. Chen YS, Lee GC, Shaw JF. 2006. Gene cloning, expression, and biochemical characterization of a recombinant trehalose synthase from *Picrophilus torridus* in *Escherichia coli*. *J Agric Food Chem* 54:7098-7104.
15. Yue M, Wu XL, Gong WN, Ding HB. 2009. Molecular cloning and expression of a novel trehalose synthase gene from *Enterobacter hormaechei*. *Microb Cell Factories* 8 :1-7.
16. Lee JH, Lee KH, Kim CG, Lee SY, Kim GJ, Park YH, Chung SO. 2005.Cloning and expression of a trehalose synthase from *Pseudomonas stutzeri* CJ38 in Escherichia coli for the production of trehalose. *Appl Microbiol Biotechnol* 68: 213-219.
17. Li Y, Sun X, Feng Y, Yuan Q. 2015. Cloning, expression and activity optimization of trehalose synthase from *Thermus thermophilus* HB27. *Chem Eng Sci* 135:323-329.
18. Nishimoto T, Nakano M, Nakada T, Chaen H, Fukuda S, Sugimoto T, Tsujisaka Y. 1996. Purification and characterization of a thermostable trehalose synthase from *Thermus aquaticus*. *Biosci Biotechnol Biochem* 60: 835-839.
19. Koh SK, Shin HJ, Kim JS, Lee DS, Lee SY. 1998. Trehalose synthesis from maltose by a thermostable trehalose synthase from *Thermus caldophilus*. *Biotechnol Lett* 20: 757-761.
20. Koh S, Kim J, Shin HJ, Lee D, Bae J, Kim D, Lee DS. 2003. Mechanistic study of the intramolecular conversion of maltose to trehalose by *Thermus caldophilus* GK24 trehalose synthase. *Carbohydr Res* 338: 1339−1343.
21. Jiang L, Lin M, Zhang Y, Li Y, Xu X, Li S, Huang H. 2013. Identification and characterization of a novel trehalose synthase gene derived from saline-alkali soil metagenomes. *PloS one 8*: e77437.
